# The endoscope-assisted supraorbital “keyhole” approach for anterior skull base meningiomas: an updated meta-analysis

**DOI:** 10.1007/s00701-020-04544-x

**Published:** 2020-09-05

**Authors:** Danyal Z. Khan, Ivo S. Muskens, Rania A. Mekary, Amir H. Zamanipoor Najafabadi, Adel E. Helmy, Robert Reisch, Marike L. D. Broekman, Hani J. Marcus

**Affiliations:** 1 Division of Neurosurgery, Department of Clinical Neurosciences, University of Cambridge, Cambridge, UK; 2 Department of Neurosurgery, Haaglanden Medical Center and Leiden University Medical Center, The Hague, The Netherlands; 3 Computational Neurosciences Outcomes Center, Department of Neurosurgery, Brigham and Women’s Hospital, Boston, MA USA; 4 Department of Pharmaceutical Business and Administrative Sciences, School of Pharmacy, MCPHS University, Boston, MA USA; 5 Department of Neurosurgery, University Neurosurgical Centre Holland, Leiden University Medical Centre, Haaglanden Medical Centre and Haga Teaching Hospital, Leiden, and The Hague, The Netherlands; 6 ENDOMIN - Center for Endoscopic and Minimally Invasive Neurosurgery, Hirslanden Hospital, Zurich, Switzerland; 7 Department of Neurology, Massachusetts General Hospital, Harvard Medical School, Boston, MA USA; 8 Department of Neurosurgery, National Hospital for Neurology and Neurosurgery, London, UK; 9 Wellcome/EPSRC Centre for Interventional and Surgical Sciences, University College London, London, UK

**Keywords:** Endoscopic transsphenoidal surgery, Microscopic transcranial surgery, Supraorbital keyhole, Skull base surgery, Tuberculum Sellae Meningioma, Olfactory groove meningioma

## Abstract

**Introduction:**

The gold-standard treatment for symptomatic anterior skull base meningiomas is surgical resection. The endoscope-assisted supraorbital “keyhole” approach (eSKA) is a promising technique for surgical resection of olfactory groove (OGM) and tuberculum sellae meningioma (TSM) but has yet to be compared with the microscopic transcranial (mTCA) and the expanded endoscopic endonasal approach (EEA) in the context of existing literature.

**Methods:**

An updated study-level meta-analysis on surgical outcomes and complications of OGM and TSM operated with the eSKA, mTCA, and EEA was conducted using random-effect models.

**Results:**

A total of 2285 articles were screened, yielding 96 studies (2191 TSM and 1510 OGM patients). In terms of effectiveness, gross total resection incidence was highest in mTCA (89.6% TSM, 91.1% OGM), followed by eSKA (85.2% TSM, 84.9% OGM) and EEA (83.9% TSM, 82.8% OGM). Additionally, the EEA group had the highest incidence of visual improvement (81.9% TSM, 54.6% OGM), followed by eSKA (65.9% TSM, 52.9% OGM) and mTCA (63.9% TSM, 45.7% OGM). However, in terms of safety, the EEA possessed the highest cerebrospinal fluid leak incidence (9.2% TSM, 14.5% OGM), compared with eSKA (2.1% TSM, 1.6% OGM) and mTCA (1.6% TSM, 6.5% OGM). Finally, mortality and intraoperative arterial injury were 1% or lower across all subgroups.

**Conclusions:**

In the context of diverse study populations, the eSKA appeared not to be associated with increased adverse outcomes when compared with mTCA and EEA and offered comparable effectiveness. Case-selection is paramount in establishing a role for the eSKA in anterior skull base tumours.

**Electronic supplementary material:**

The online version of this article (10.1007/s00701-020-04544-x) contains supplementary material, which is available to authorized users.

## Introduction

The gold standard treatment for symptomatic anterior skull base meningiomas is complete surgical resection—if possible to do so without causing significant morbidity. Although the traditional microscopic transcranial approach (mTCA) has proven to be effective at removing such tumours [84, 86], minimally invasive surgical approaches may offer the possibility of reducing brain exposure and manipulation, and therefore increasing safety [105]. However, these less invasive techniques are often technically challenging with steep learning curves [105]. Factors influencing case-by-case surgical decision-making include the preservation of olfaction and vision, tumour size and location, the involvement of neurovascular structures, surgical experience, and patient choice [24, 86].

A previous comprehensive meta-analysis comparing the traditional mTCA and the expanded endoscopic endonasal approach (EEA) found similar gross total resection (GTR) and mortality rates, with more favourable visual outcomes but higher cerebrospinal (CSF) leak incidence with EEA [84]. This generally corroborates with findings from other systematic reviews in the field [24, 59, 110]. However, a third approach—the endoscope-assisted supraorbital “keyhole” approach (eSKA)—has yet to be compared with mTCA and EEA in the context of existing literature. This approach includes multiple variations (such as the medial supraorbital, basal supraorbital, and lateral supraorbital approaches) that are unified by the principle of achieving surgical control of a deep-seated lesion whilst minimizing iatrogenic injury to the brain (via exposure, retraction, and manipulation) [102, 107]. This is achieved through using smaller craniotomies with smaller dural openings and may theoretically reduce post-operative complications and length of stay, whilst improving cosmesis, patient satisfaction and carrying lower CSF leak rates than the EEA [102, 104, 105, 107]. However, important limitations of the eSKA include (a) difficult visualization and orientation of deep structures, (b) difficult (almost co-axial) instrument control owing to instrument size and the fulcrum effect (requiring specialized instruments), and (c) limited and predefined surgical corridors which require extensive pre-operative planning [102, 107]. Endoscope assistance provides a high light intensity with wider viewing angles distal to the craniotomy, allowing high-resolution visualization of deeper tissues. Indeed, combined with image-guidance systems and intra-operative adjuncts (e.g. ultrasound, MRI), endoscopes facilitate surgical orientation and resection during keyhole approaches [102, 107].

Therefore, we updated a previous systematic review and meta-analysis comparing mTCA with EEA and extended this review with the eSKA for the management of olfactory groove (OGM) and tuberculum sellae meningiomas (TSM).

## Methods

In order to identify studies reporting on outcomes of surgically treated TSMs and OGMs, we adapted our previous methodology [84], expanding our search to include eSKA and updating our search to include mTCA and EEA articles published after our last search.

### Search strategy

This review was conducted in accordance with the Preferred Reporting Items for Systematic Reviews and Meta-Analyses (PRISMA) Statement [81]. A search strategy was created using the keywords “Meningioma,” “Tuberculum Sellae,” “Olfactory Groove,” and synonyms (Appendix A). Studies were included if they reported on (1) patients with olfactory groove (OGM) or tuberculum sellae (TSM) meningiomas; (2) patients undergoing surgery using the mTCA, EEA or eSKA approaches; and (3) surgical outcomes and complications. Exclusion criteria included case reports, commentaries, congress abstracts, reviews, animal studies, studies describing a combined surgical approach (for example EEA + mTCA), studies in paediatric patients (< 18 years old), re-operations, and cadaveric studies. A date filter was applied, with articles from 2004 to 2020 being included—reflecting a period of the contemporary adaptation of endoscopic and keyhole approaches and the continuous improvement of traditional microsurgical approaches for the relevant pathologies [11, 15, 32, 106].

Both PubMed and Embase databases were searched on 19 April 2020. Duplicates were removed using Endnote X9. Independent title and abstract screening of updated results was performed in duplicate by two authors (DZK, HJM). Review of full-text articles ensued according to the inclusion/exclusion criteria. Any discrepancies in selection were settled out by discussion and mutual agreement.

### Data extraction

Data points extracted from the included articles comprised of patient characteristics (age, sex distribution), tumour characteristics (surgical approach used, sum of sample, tumour grade, tumour diameter or volume, follow-up length), outcomes (GTR, visual improvement), and complications (CSF leak, 30-day mortality, intra-operative arterial injury). World Health Organization (WHO) grading included recording the proportion of WHO Grade 1 tumours [72]. Of note, the grading system was revised in 2016 to include brain invasion as a criterion to upgrade Grade 1 tumours to Grade 2 [72]. Therefore, if any studies pre-2016 reported brain invasion amongst Grade 1 tumours, the respective tumours were upgraded accordingly [72]. Gross total resection (GTR) referred to Simpson Grades 1 and 2 as per our original methodology [84, 116]. Visual improvement was in the context of those with preoperative visual problems only. Mortality (within 30 days after resection) was recorded on an all-cause basis.

Owing to the not uncommon reporting of follow-up time as a median number of months (as opposed to mean), the estimated mean number of months was calculated as per recommendations of Hozo et al. [53]. Of note, in sample sizes greater than 25, the sample’s median follow-up is presented as the best estimate of the mean [53].

Importantly, studies that did not report specific outcomes for OGM/TSM and approach combination were excluded from the final meta-analysis. These studies were considered for qualitative analysis if the relevant the tumour (TSM or OGM) and approach (mTCA, EEA, or eSKA) combination of interest was > 50% of the study population [9, 48, 98, 105, 111]. Similarly, articles that grouped TSM cases with planum sphenoidale meningiomas [3, 92] were considered for qualitative review only (owing to the similarity of these tumour groups) but not included in the final meta-analysis.

### Risk of bias assessment

Study quality was assessed with a modified New-Castle Ottawa Scale (mNOS), which assesses two domains: sample selection and outcome reporting. The modification made to the original NOS was the exclusion of the “comparability” domain as this is not applicable to case series [126]. The scale is scored out of 6 (3 for selection domain, 3 for outcome domain). Publication bias was assessed using Begg’s tests [8] and by generating funnel plots with and without trim-and-fill method [34].

### Meta-analysis

A meta-analysis was conducted using R 3.6.1 (The R Foundation, Austria) applying the “meta” package. Pooled incidence (using the random-effect model method of DerSimonian and Laird [33]) was calculated for each approach (eSKA, mTCA, EEA), tumour (TSM, OGM), and outcome (GTR, arterial injury, visual improvement, CSF leakage, and mortality) combination. Study heterogeneity was assessed by calculating *I*-squared values [52] (*I*^2^ > 50% considered significant) and Cochran’s *Q* test (*p* < 0.10) [36, 52]. Sensitivity analysis was performed by running the above analyses on a low risk of bias sub-group (mNOS score greater than or equal to 4).

Additionally, a univariate meta-regression was performed to explore the effect of mean age (continuous variable) and male percentage (continuous variable) on each approach, tumour, and outcome combination. Meta-regression was only performed if 8 or more studies were available for the outcome/approach/tumour combination being explored. This threshold was chosen (a deviation from the standard threshold of 10) on a pragmatic basis, to reflect the relative paucity of literature from the newer eSKA approach [51]. This threshold was also applied to the performance of Begg’s test, trim-and-fill analysis, and the generation of funnel plots.

## Results

### Search results

**Fig. 1:**
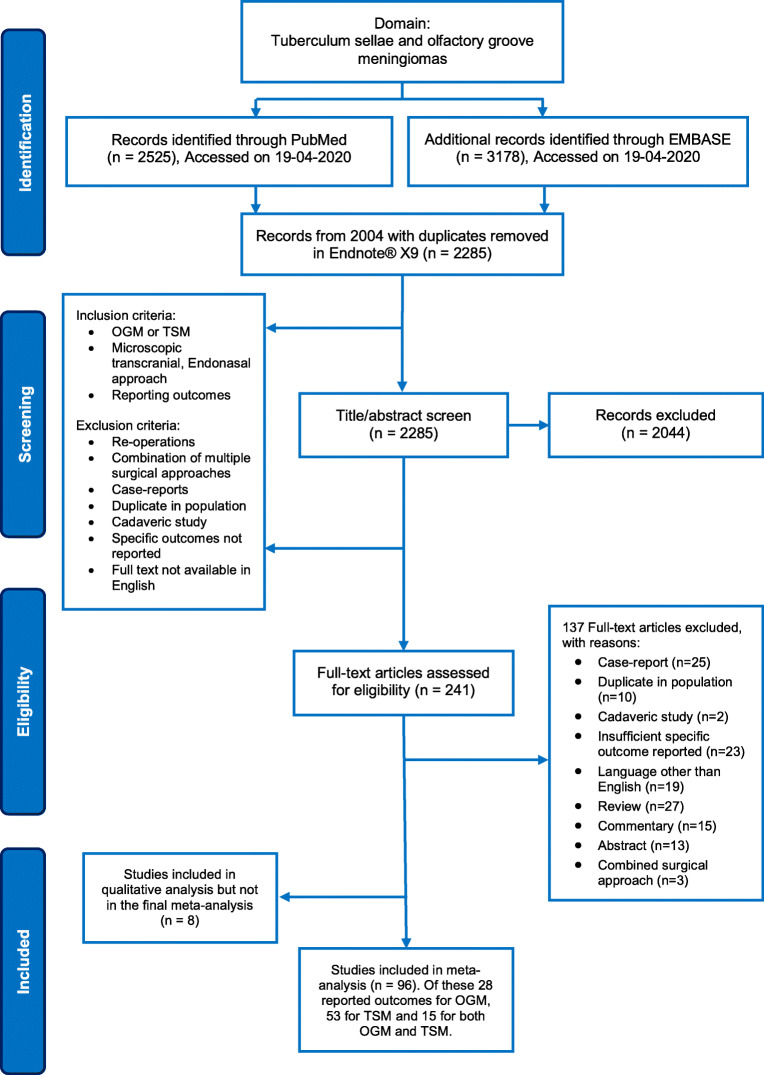
PRISMA flowchart detailing search strategy and systematic article selection

In all, after removing duplicates, 2285 articles were identified (Fig. 1). After screening for titles and abstracts, 2044 articles were excluded and 241 full texts were reviewed to yield 96 included studies. Fifty-three TSM-only case series were included in the meta-analysis of which 21 involved the EEA [3, 12, 13, 17, 19, 22, 37, 41, 49, 60, 61, 63, 67, 74, 91, 92, 120, 125, 131], 37 the mTCA [1, 3, 6, 13, 20, 21, 26, 29, 38, 43, 45, 55, 60, 63, 64, 66, 68, 74–76, 78, 79, 82, 85, 88, 92, 95, 97, 98, 108, 112, 114, 120, 124, 127, 130], and 5 the eSKA [16, 35, 41, 67, 78] with 10 of these papers covering multiple approaches [3, 14, 41, 60, 63, 67, 74, 78, 92, 120]. Twenty-eight OGM-only case series were included in the meta-analysis of which 5 involved EEA [4, 28, 62, 70, 92], 24 in mTCA [5, 7, 10, 23, 25, 27, 28, 40, 42, 44, 47, 56, 57, 70, 83, 87, 89, 96, 99, 101, 109, 117, 121, 123], 3 in eSKA [4, 39, 92], and with 4 of these studies detailing multiple approaches [4, 28, 70, 92]. Additionally, 15 studies explored both OGM and TSM [9, 30, 31, 50, 54, 58, 65, 93, 94, 103, 105, 113, 122, 128, 129]. Resultantly, the TSM group totalled 2191 patients and OSM group totalled 1519 patients (Tables 1 and 2).

**Table 1:** Summary study characteristics for tuberculum sellae meningioma papers. WHO: World Health Organisation, mNOS: *modified Newcastle Ottawa Score*

	Endoscopic endonasal approach		Endoscope-assisted supraorbital keyhole approach		Microscopic transcranial approach	
	Amount	Data unavailable	Amount	Data unavailable	Amount	Data unavailable
Aggregate number of studies	26	*-*	11	-	42	*-*
Total number of patients	540	*-*	128	*-*	1523	*-*
Median mean age (years)	54.4	4 studies	57	1 study	53.8	8 studies
Median male %	25%	4 studies	16.7%	2 studies	23.4%	5 studies
Median number of WHO grade 1	20	15 studies	11.5	5 studies	26.5	26 studies
Median mean tumour diameter (cm)	2.5 (7 studies)	9 studies	2.9 (2 studies)	2 studies	2.5 (17 studies)	17 studies
Median mean tumour volume (cm^3^)	6.1 (10 studies)		12.4 (7 studies)		8.2 (8 studies)	
Median mean follow-up (months)	27	6 studies	39.8	1 study	39.5	5 studies
Median mNOS score	4	*-*	5	-	4	*-*

**Table 2:** Summary study characteristics for olfactory groove meningioma papers. WHO: World Health Organisation, mNOS: modified Newcastle Ottawa Score

	Endoscopic endonasal approach		Endoscope-assisted supraorbital keyhole approach		Microscopic transcranial approach	
	Amount	Data unavailable	Amount	Data unavailable	Amount	Data unavailable
Aggregate number of studies	10	*-*	9	-	29	*-*
Total number of patients	115	*-*	96	*-*	1308	*-*
Median mean age (years)	53.1	1 studies	59.2	1 studies	54	4 studies
Median male %	22.5%	2 studies	57.1%	2 studies	32.4%	3 studies
Median number of WHO grade 1	9	5 studies	8.5	7 studies	48	13 studies
Median mean tumour diameter (cm)	4 (1 study)	4 studies	NA	3 studies	4.6 (15 studies)	10 studies
Median mean tumour volume (cm^3^)	33.3 (5 studies)		24.8 (6 studies)		42.5 (4 studies)	
Median mean follow-up (months)	35.3	2 studies	5	1 studies	54	1 studies
Median mNOS score	4.5	*-*	45.1	-	4	*-*

### General characteristics

The median number of patients per study was 20 (range: 3–95) for TSM and 19.5 (range: 2–129) for OGM. The average percentage of male patients was 24% for TSM and 31% for OGM. The median mean of age was 54.2 years for TSM and 54.75 years for OGM. The median mean of follow-up time for TSM was 32 months (reported in 55/67 studies) for and 44.5 months for OGM studies (reported in 39/43 studies). The modified NOS score varied between 2/6 and 6/6 amongst the TSM and OGM case series, with predominant factors affecting this variance being a description of follow-up and outcome reporting. Summary characteristics by approach (eSKA, EEA, or mTCA) are highlighted in Tables 1 and 2. Individual study characteristics are displayed in Tables 5 and 6 (Appendix B).

### Gross total resection

#### Tuberculum sellae meningioma

**Fig. 2:**
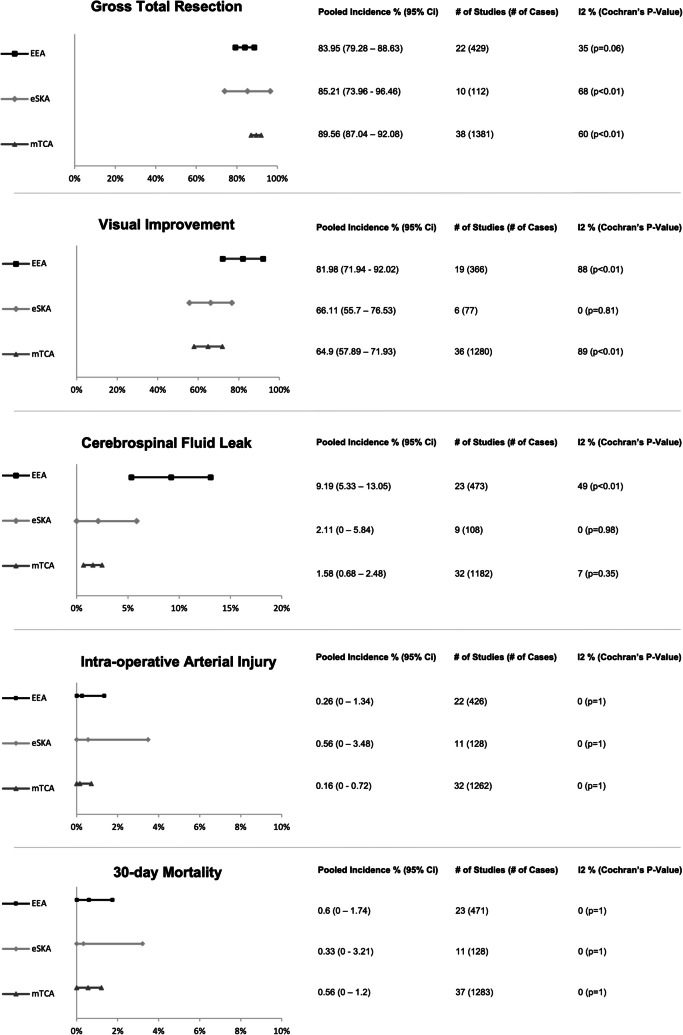
Graphical display of pooled random effects per outcome metric for Tuberculum Sellae Meningioma. EEA: Expanded endonasal approach, eSKA: Endoscope assisted supra-orbital keyhole approach, mTCA: Microscopic transcranial approach, CI: Confidence Interval

GTR was reported in 10 eSKA (112 patients), 22 EEA (429 patients), and 38 mTCA (1381 patients) studies. Pooled incidence of GTR (Fig. 2; Appendix C) was highest in the mTCA group at 89.56% (95% CI 87.04–92.08) followed by eSKA at 85.21% (95% CI 73.96–96.46) and EEA at 83.95% (95% CI 79.28–88.63). Study heterogeneity was significant within the eSKA (*I*^2^ = 68%, Cochran’s *p* < 0.01) and mTCA (I^2^ = 60%, Cochran’s *p* < 0.01) groups, with Begg’s test for publication bias also significant in this mTCA group (*p* < 0.01) (Table 3). This impacted funnel plot asymmetry, which was most marked in the mTCA group, without any major change in summary effect using trim and fill across subgroups (Appendix D). Meta-regression suggests male sex was significantly associated with lower GTR incidence in EEA (slope − 0.05 (95% CI − 0.96–0.04)) and mTCA (slope − 0.27 (95% CI − 0.53 to − 0.01)) subgroups (Table 3).

**Table 3:** Outcomes of the tuberculum sellae meningioma (TSM)—meta-regression based on age and male percentage. CI – confidence interval, NA – not available

Outcomes in TSM	Begg’s test (*p*-value)	Meta-regression on age slope (95% CI)	Meta-regression on age (*p*-value)	Meta-regression on sex slope (95% CI)	Meta-regression on sex (*p*-value)
Gross total resection (Simpson Grade 1 Or 2)					
Expanded endonasal approach	0.32	0.003 (− 0.006–0.01)	0.51	− 0.5 (− 0.96–0.04)	0.33
Endoscope-assisted supraorbital keyhole approach	0.32	0.03 (− 0.01–0.06)	0.06	− 0.75 (− 1.75–0.26)	0.14
Microscopic transcranial approach	< 0.01	0.001 (− 0.005–0.007)	0.75	− 0.27 (− 0.53 - − 0.01)	0.04
Visual improvement					
Expanded endonasal approach	0.67	− 0.005 (− 0.01–0.005)	0.36	− 0.38 (− 0.82–0.06)	0.09
Endoscope-assisted supraorbital keyhole approach	NA	NA	NA	NA	NA
Microsopic transcranial approach	0.35	− 0.005 (− 0.02–0.01)	0.57	0.11 (− 0.68–0.91)	0.78
Cerebrospinal fluid leak					
Expanded endonasal approach	0.03	− 0.001 (− 0.008–0.008)	0.95	− 0.04 (− 0.47–0.39)	0.86
Endoscope-assisted supraorbital keyhole approach	0.21	− 0.001 (− 0.01–0.01)	0.83	− 0.08 (− 0.47–0.31)	0.7
Microscopic transcranial approach	< 0.01	0.001 (− 0.003–0.004)	0.75	0.07 (− 0.04–0.18)	0.23
Intra-operative arterial injury					
Expanded endonasal approach	< 0.01	0.001 (− 0.004 to − 0.004)	0.88	− 0.02 (− 0.21–0.17)	0.84
Endoscope-assisted supraorbital keyhole approach	0.01	− 0.001 (− 0.008–0.008)	0.95	0.01 (− 0.33–0.35)	0.95
Microsopic transcranial approach	< 0.01	− 9.53 (− 0.002–0.002)	0.91	− 0.006 (− 0.07–0.06)	0.87
30-day mortality					
Expanded endonasal approach	< 0.01	0.002 (− 0.003–0.006)	0.48	0.04 (− 0.18–0.26)	0.74
Endoscope-assisted supraorbital keyhole approach	< 0.01	0.001 (− 0.007–0.009)	0.87	− 0.02 (− 0.36–0.3)	0.87
Microsopic transcranial approach	< 0.01	0.001 (− 0.001–0.002)	0.57	− 0.001 (− 0.07–0.07)	0.99

#### Olfactory groove meningioma

**Fig. 3:**
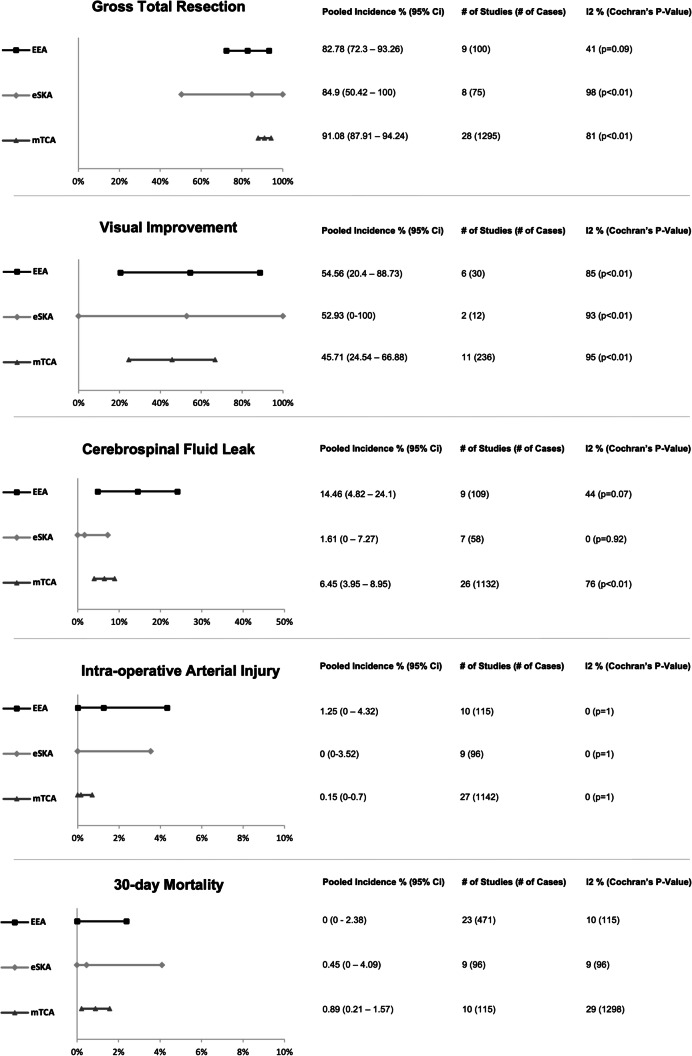
Graphical display of pooled random effects per outcome metric for Olfactory Groove Meningioma. EEA: Expanded endonasal approach, eSKA: Endoscope assisted supra-orbital keyhole approach, mTCA: Microscopic transcranial approach, CI: Confidence Interval

GTR incidence was reported in 8 eSKA (75 patients), 9 mTCA (100 patients), and 28 mTCA (1295 patients) studies. The pooled incidence of GTR (Fig. 3; Appendix C) was highest in the mTCA group with 91.08% (95% CI 87.91–94.24), followed by eSKA with 84.9% (95% CI 50.42–100) and EEA at 82.78% (95% CI 72.3–93.26). In terms of study heterogeneity, this was significant within the eSKA (*I*^2^ = 98%, Cochran’s *p* < 0.01) and mTCA (*I*^2^ = 81%, Cochran’s *p* <0.01) groups, with Begg’s test for publication bias also significant in this mTCA group (*p* < 0.01) (Table 4). These findings are similar to those of the TSM group. Funnel plot asymmetry was most marked in mTCA (reflective of heterogeneity and publication bias) and eSKA (likely reflective of heterogeneity groups). There was no major change in summary effect using trim-and fill-method across subgroups (Appendix D). In the eSKA subgroup, older age was associated with increased GTR on meta-regression (slope 0.05 (95% CI 0.02–0.08)) (Table 4).

**Table 4:** Outcomes of the olfactory groove meningioma (OGM): Meta-regression based on age and male percentage. CI - confidence interval, NA – Not available

Outcomes in OGM	Begg’s test (*p*-value)	Meta-regression on age slope (95% CI)	Meta-regression on age (*p*-value)	Meta-regression on sex slope (95% CI)	Meta-regression on sex (*p*-value)
Gross total resection (Simpson Grade 1 Or 2)					
Expanded endonasal approach	1	− 0.01 (− 0.02–0.01)	0.45	− 0.3 (− 1.34–0.74)	0.58
endoscope-assisted supraorbital keyhole approach	0.51	0.05 (0.02–0.08)	< 0.01	− 0.28 (− 2.29–1.71)	0.78
Microsopic transcranial approach	0.01	0.01 (− 0.01–0.01)	0.49	− 0.01 (− 0.29–0.28)	0.96
Visual improvement					
Expanded endonasal approach	0.04	− 5.07 (− 0.04–0.04)	0.99	0.3 (− 3.3–3.9)	0.87
Endoscope-assisted supraorbital keyhole approach	NA	NA	NA	NA	NA
Microsopic transcranial approach	0.48	0.03 (− 0.06–0.12)	0.55	− 0.47 (− 3.25–2.3)	0.74
Cerebrospinal fluid leak					
Expanded endonasal approach	0.64	− 0.002 (− 0.02–0.01)	0.85	0.79 (0.2–1.38)	0.01
Endoscope-assisted supraorbital keyhole approach	NA	NA	NA	NA	NA
Microsopic transcranial approach	0.01	− 0.002 (− 0.009–0.005)	0.51	0.001 (− 0.23–0.23)	0.99
Intra-operative arterial injury					
Expanded endonasal approach	0.02	0.001 (− 0.001–0.01)	0.86	0.08 (− 0.29–0.44)	0.68
Endoscope-assisted supraorbital keyhole approach	NA	0 (− 0.008–0.008)	1	0 (− 0.24–0.24)	1
Microsopic transcranial approach	0.01	− 4.56 (− 0.002–0.001)	0.95	− 0.01 (− 0.08–0.05)	0.68
30-day mortality					
Expanded endonasal approach	0.01	0 (− 0.01–0.01)	1	0 (− 0.35–0.35)	1
Endoscope-assisted supraorbital keyhole approach	0.01	− 0.001 (− 0.008–0.007)	0.97	− 0.06 (− 0.33–0.21)	0.66
Microsopic transcranial approach	0.01	− 0.001 (-0.003–0.001)	0.07	− 0.09 (− 0.16–0.02)	0.01

### Visual improvement

#### Tuberculum sellae meningioma

Pre-operative visual impairment was reported in 6 eSKA (77 patients), 19 EEA (366 patients), and 36 mTCA (1280 patients) studies. The pooled incidence of visual improvement (Fig. 2; Appendix C) in the EEA group was 81.98% (95% CI 71.94–92.02) and was higher than the eSKA at 65.98% (95% CI 54.4–77.56) and mTCA at 63.9% (95% CI 57.15–70.65). However, study heterogeneity was significant within the EEA (*I*^2^ = 88%, Cochran’s *p* < 0.01) and mTCA (*I*^2^ = 89%, Cochran’s *p* < 0.01) groups. Publication bias was not evident on Begg’s testing, with mild funnel plot asymmetry in mTCA and EEA groups likely due to heterogeneity. This is supported by the lack of its major change in summary effect using trim-and-fill across subgroups (Appendix D). Meta-regression on age and sex did not reach statistical significance across mTCA, EEA, and eSKA groups (Table 3).

#### Olfactory groove meningioma

Pre-operative visual impairment was reported in 2 eSKA (12 patients), 6 EEA (30 patients), and 11 mTCA (236 patients) studies. The pooled incidence of visual improvement (Fig. 3; Appendix C) in descending order were as follows: EEA at 54.56% (95% CI 20.4–88.73), eSKA at 52.93% (95% CI 0–100) and mTCA with 45.71% (95% CI 24.54–66.88)—a similar pattern to the TSM group. Study heterogeneity was significant across all subgroups: EEA (*I*^2^ = 85%, Cochran’s *p* < 0.01), eSKA (*I*^2^ = 93%, Cochran’s *p* < 0.01), and mTCA (*I*^2^ = 95%, Cochrans *p* < 0.01). Publication bias was evident in the EEA cohort (Begg test, *p* = 0.04), with both this and the above heterogeneity contributing to the marked funnel plot asymmetry (Appendix D). Using the trim and fill method does not display a marked difference in summary effects (Appendix D). Meta-regression on age and sex did not reach statistical significance across subgroups (Table 4).

### Cerebrospinal fluid leakage

#### Tuberculum sellae meningioma

Incidence of postoperative CSF leakage was reported in 9 eSKA (108 patients), 23 EEA (473 patients), and 32 mTCA (1182 patients) studies. The pooled incidence of CSF leak (Fig. 2; Appendix C) in the EEA group was 9.19% (95% CI 5.33–13.05), which was higher than the incidence observed among the eSKA treated group at 2.11% (95% CI 0–5.84) and mTCA treated group at 1.58% (95% CI 0.68–2.48). However, study heterogeneity (*I*^2^ = 49%, Cochran’s *p* < 0.01) and publication bias (Begg’s p=0.03) were significant in the EEA group. Publication bias was also evident in the mTCA group (Begg’s *p* ≤ 0.01). The asymmetry of mTCA and EEA funnel plots is explained by the above (Appendix D), but no major change in summary effect using the trim-and-fill method is appreciable in these groups. Meta-regression on age and sex did not reach statistical significance across any group (Table 3).

#### *Olfactory groove meningioma*

Incidence of post-op CSF leakage was reported in 7 eSKA (58 patients), 9 EEA (109 patients), and 26 mTCA (1132 patients) studies. Pooled incidence of CSF leak (Fig. 3; Appendix C) in the EEA group was 14.46% (95% CI 4.82–24.1), 6.45% in the mTCA group (95% CI 3.95–8.95), and 1.61% (95% CI 0–7.27) in the eSKA group. Study heterogeneity was evident in the mTCA group (I^2^ = 76%, Cochran’s *p* < 0.01), whilst publication bias was suggested in the mTCA (Begg’s *p* ≤ 0.01) and eSKA (Begg’s *p* = 0.03). Indeed, mTCA and eSKA funnel plots reflect this in their asymmetry (Appendix D). Meta-regression suggested male sex was associated with increased CSF leak in the EEA approach (slope 0.79 (95% CI 0.2–1.38)) (Table 4).

### Intraoperative arterial injury

#### Tuberculum sellae meningioma

Incidence of intraoperative arterial injury was reported in 11 eSKA (128 patients), 22 EEA (426 patients), and 32 mTCA (1262 patients) studies. Pooled incidence (Fig. 2; Appendix C) in descending order were as follows: eSKA − 0.56% (95% CI 0–3.48), EEA − 0.26% (95% CI 0–1.34), and mTCA − 0.16% (95% CI 0–0.72). Across all 3 groups, study heterogeneity was not apparent; however, publication bias using Begg’s test reached statistical significance in eSKA (*p* = 0.01), EEA (*p* < 0.01), and mTCA (*p* < 0.01) groups—explaining funnel plot asymmetry across groups. Trim and fill adjustment, however, made almost no difference in overall summary effects (Appendix D). Meta-regression did not reveal significant associations for age and sex across all treatment groups (Table 3).

#### Olfactory groove meningioma

Incidence of intraoperative arterial injury was reported in 9 eSKA (96 patients), 10 EEA (115 patients), and 27 mTCA (1142 patients) studies. Pooled incidence (Fig. 3; Appendix C) was highest in the EEA group at 1.25% (95% CI 0–4.32), followed by the mTCA at 0.15% (95% CI 0–0.7) and eSKA with 0% (95% CI 0–3.52). Indeed, these results do not align with the TSM group. Across all 3 groups, study heterogeneity was not apparent; however, publication bias using Begg’s test reached statistical significance in EEA (*p* = 0.02) and mTCA (*p* < 0.01) groups, mapping to funnel plot asymmetry in EEA and mTCA groups. However, trim and fill adjustment made only minor differences to the estimated summary effect (Appendix D). Again, meta-regression did not show a significant effect of age and sex on arterial injury (Table 4).

### 30-day mortality

#### Tuberculum sellae meningioma

Incidence of mortality was reported in 11 eSKA (128 patients), 23 EEA (471 patients), and 37 mTCA (1283 patients) studies. Pooled incidence of 30-day mortality (Fig. 2; Appendix C) was 0.6% (95% CI 0–1.74) in the EEA group, followed by 0.56% (95% CI 0–1.2) in mTCA and 0.33% (95% CI 0–3.21) in eSKA in descending order. Across all 3 groups, study heterogeneity was not apparent; however, publication bias using Begg’s test reached statistical significance in all three groups (*p* < 0.01). Resultantly, the mTCA and eSKA funnel plots are asymmetrical but are not appreciably impacted in terms of summary effect by the implementation of trim and fill (Appendix D). Meta-regression did not show a significant effect of age and sex on mortality across subgroups (Table 3).

#### Olfactory groove meningioma

Incidence of mortality was reported in 9 eSKA (96 patients), 10 EEA (115 patients), and 23 mTCA (471 patients) studies. Unlike, the TSM population, pooled incidence of 30-day mortality (Fig. 3; Appendix C) was greatest in the mTCA group at 0.89% (95% CI 0.21–1.57), followed by the eSKA at 0.45% (95% CI 0–4.09) and EEA with 0% (95% CI 0–2.38). Across all 3 groups, study heterogeneity was not apparent; however, publication bias using Begg’s test reached statistical significance in all groups (*p* < 0.1), mapping to funnel plot asymmetry in EEA and mTCA groups (Appendix D). Trim and fill implementation did not result in any major adjustment to the estimated summary effect. Male sex appeared to be associated with higher 30-day mortality in the mTCA (slope − 0.09 (95% CI − 0.016–0.02)) (Table 4).

### Sensitivity analysis with low risk of bias studies

The pooled incidence of surgical outcomes of a subgroup of low-risk studies (defined as mNOS score greater than or equal to 4) is presented in Appendix E. This analysis, when compared with the total group analysis, yielded overall similar results for GTR, mortality, and intraoperative arterial injury. CSF leak incidence after EEA was apparently lower (in both OGM and TSM), and visual improvements after EEA (in the TSM group) were more marked in the lower risk of bias studies.

## Discussion

### Principle findings

In the context of heterogeneous study populations and outcome reporting, the endoscope-assisted supraorbital “keyhole” approach appeared to be associated with similar effectiveness (GTR, visual improvement) and safety (CSF leak, 30-day mortality) compared with the mTCA and EEA alternatives based on our findings. Case selection and an understanding of relative indications are important in selecting the most appropriate approach for anterior skull base meningioma resection.

As previously found, the EEA was associated with the highest rates of visual improvement across OGM and TSM groups. However, this advantage of EEA may be offset when considering the safety profile of the three approaches, with the EEA having the highest incidence of post-op CSF leak (statistically significant in the TSM sub-group). In contrast, the mTCA had a slightly higher incidence of GTR than eSKA and EEA (eSKA > EEA) across TSM and OGM groups. Interestingly, the eSKA displays intermediate results in terms of efficacy (GTR and visual improvement) and complications (CSF leak). Results for intra-operative arterial injury and 30-day mortality incidences are similar and overlapping across the 3 approaches. Indeed, the eSKA, as a relatively new technique, is less well explored. When compared with the mTCA, the eSKA—as a minimally invasive technique—offers a smaller craniotomy scar, less brain exposure, and less brain/nerve retraction [105]. Thus, theoretically, it shares similar limitations to the minimally invasive EEA [92]—potentially making total resection of larger tumours or tumours with significant local invasion difficult [24, 92, 105]. However, when performed with the benefit of neuronavigation, neuroendoscopy (12/13 of eSKA studies in our meta-analysis), and appropriate surgical training, the eSKA has been used to resect relatively large tumours of the anterior skull base [4, 41, 67, 105].

All 3 approaches likely have their own role in the management of anterior skull base meningiomas, with their varying safety and efficacy profiles as evidenced above. Case selection will be paramount in establishing a role for each technique/combination of techniques [4, 92, 105]. Indeed, case selection of eSKA is currently considerably variable, owing to its novelty and ongoing refinement [70, 92]. The selection of the preferred approach for each case must be taken in the context: (a) patient-related factors (demographic, presentation, preferences), (b) tumour-related factors (size, consistency, extension, location—such as relation to optic foramen or cribriform plate), and (c) surgeon experience, surgeon preference, and surgical goals (such as GTR or STR, visual or olfactory preservation) [2, 77, 92, 105, 118]. Ottenhausen et al. presents a concise decision-making algorithm (based on tumour anatomy and resulting functional deficits), which incorporates the specific characteristics of eSKA, EEA, and mTCA approaches [92]. In this algorithm, the eSKA is suitable for TSMs with lateral extension up to the internal carotid arteries (ICA) and anterior clinoid processes (ACP), or lateral extension beyond the lamina papyracea (LP). Additionally, the eSKA is suggested for OGMs with (1) preserved olfaction and (2) disrupted olfaction without cribriform plate invasion but with significant anterior (up to the frontal sinus) or lateral extension. In contrast, the EEA is proposed for TSM without significant lateral extension (ICA/ACP/LP as above) and OGMs without significant lateral extension (where olfaction is disrupted). Finally, an mTCA or a combined EEA + eSKA approach is suggested for OGMs and TSMs with a significant anterior or lateral extension (unless there is no cribriform plate invasion, in which case, eSKA alone may be possible) [92]. Of note, other algorithms cite > 5mm sellar extension and optic canal involvement as factors favouring EEA in TSM [60]. During EEA for TSM, decompression of the optic canal from below avoids excessive vascular manipulation, can be achieved before or after tumour resection, and is well suited to tumours with extension into the inferomedial aspect of the optic canal [2, 69]. Decompression of the involved optic canal is described in mTCA approaches with early decompression (before tumour resection) favoured [20, 80, 90]. In eSKA, studies describe both early and late bilateral canal decompression with optimum timing being less clear [16, 67]. More generally, within the literature, consensus for the ideal surgical approach in various contexts is not clear [41, 60, 67, 74, 92, 99]. Indeed, in light of the COVID-19 pandemic—which elucidated to the risk endonasal surgery may pose (exposing theatre staff to high viral loads and potentially serious infection)—this case selection process is likely to be a dynamic field in the near future [71, 100, 119].

### Findings in the context of other syntheses

Previous meta-analyses have compared the EEA and mTCA (not eSKA) with varying results.

In terms of GTR, Muskens et al. (co-author) previously found higher GTR incidence with mTCA in OGM at 88.5% (CI 85.9–90.7%) versus EEA 70.9% (CI 60.3–79.9%) [84]—in line with our findings. This corroborates with other meta-analyses. Ruggeri et al. explored OGM and TSM, finding a higher GTR rate (*p* < 0.01) in mTCA (88.13%) than EEA (78,42%) [110]. Similarly, Komotor et al. highlighted a 92.8% GTR rate in mTCA, compared with 63.2% in EEA (0.001) in the context of TSM and OGM [59], whilst Shetty et al. explored GTR in OGM, finding a significantly (*p* < 0.01) higher rate in mTCA (90.9%) than in EEA (70.2%) [115].

Regarding visual outcomes, a recent comparative meta-analysis by Lu et al. suggests improved visual outcome in OGM resection using the EEA (vs. mTCA) (OR, 0.318; *p* = 0.04) but not statistically significant in TSM [73]. This is slightly different from our updated findings and previous findings of Muskens et al. [84], in which the visual outcome advantage of EEA was most prominent in the TSM group. In other analyses, an early (2013) study by Clark et al. displayed higher (*p* < 0.01) visual improvement incidence in TSM with EEA (50–100 % in included studies) compared with mTCA studies (25–78 %) [24]. Shetty et al. explored OGM alone and found 80.7% visual improvement in the EEA studies group versus 12.83% in the mTCA group (*p* < 0.01) [115]. Ruggeri et al. replicated these findings when taking OGM and TSM as a collective group, with EEA displaying an 80.1% incidence visual improvement, significantly (*p* < 0.01) higher than mTCA (62.2%) [110].

In terms of CSF leak rate, Muskens et al. highlighted this as a disadvantage to the EEA in both TSM (EEA: 19.3% (95% CI 14.1–25.8%), mTCA 5.8% (95% CI 4.3– 7.8%)) and OGM (EEA: 25.1% (95% CI 17.5–34.8%), mTCA 10.5% (95% CI 8.2–13.4%)) [84]. This finding is echoed throughout relevant secondary literature, with Lu et al. highlighting a higher CSF leak incidence in EEA (vs mTCA) in TSM (OR 3.854; *p* = 0.013) and Shetty et al. showcasing a 25.7% CSF leak occurrence in EEA versus 6.3% in mTCA (*p* < 0.01) [73, 115]. In taking TSM and OGM, together, Komotor et al. demonstrated a higher CSF leak incidence of 21.3% in EEA, compared with 4.3% in mTCA (*p* < 0.01), whilst Ruggeri et al. illustrated 18.84% CSF leak occurrence in EEA versus 5.95% in mTCA (*p* < 0.01) [110, 115].

Finally, when considering 30-day mortality, significant associations have been difficult to establish both in our study and the literature. Ruggeri et al. found mortality rates of 2.3% in mTCA and 1.03% in EEA in TSM and OGM, but this did not reach statistical significance (*p* = 0.154) [110]. Similarly, differences in mortality explored by Muskens et al. were inconclusive in TSM (EEA: 5.2% (95% CI 3.4–10.8%), mTCA 2.7% (95% CI 1.8–4%)) and OGM (EEA: 4.3% (95% CI 1.5–11.6%), mTCA 3.9% (95% CI 2.7–5.8%)) [84]. A similar situation is found with intra-op arterial injury incidence with most syntheses not highlighting significant differences, echoed by our updated analysis [24, 59, 110, 115].

### Limitations and strengths

The principal limitations of this study are the likely prevalent publication bias and heterogeneity of the primary literature synthesized, more specifically heterogeneity in the reporting of baseline characteristics and outcomes. This is reflected in the *I*^2^ and Cochran *Q* tests highlight in Figs. 2 and 3, corroborating with respective funnel charts (Appendix B). Development of core data set, through a multi-stakeholder consensus process for example, would be useful for future pooled analysis in the field. Secondly, it is likely the study populations examined are considerably variable owing to the surgical decision-making process that informs the choice of approach. Larger, more extensive tumours may be more likely to undergo traditional open approaches (of which there are many variants) in order to achieve acceptable tumour resection [84]. This is reflected in Tables 1 and 2 where larger (diameter and/or volume) tumours are included in the mTCA group compared with the EEA group. Unfortunately, we were not able to perform meta-regression based on tumour size or grade owing to heterogeneous data reporting, potentially adding to confounding factors [18, 46]. Additionally, the overwhelming majority of studies included were case series, and thus, our interpretation of our results should be tempered to reflect this. Finally, owing to the novelty of the approach, there is a relative paucity in the amount of included eSKA studies. Although overall, the results of the main analysis are similar to that of the sensitivity analysis subgroup, the number of low risk of bias studies analyzed is also limited. Future studies in the field must improve on methodological design, with an emphasis on comparative studies, in order to facilitate more robust data synthesis.

## Conclusions

In the context of diverse study populations and heterogeneous case selection criteria, the endoscope-assisted supraorbital keyhole approach appeared not to be associated with increased adverse outcomes when compared with expanded endonasal and traditional transcranial approaches and offered comparable effectiveness. Case selection is paramount in establishing a role for the supraorbital keyhole approach in anterior skull base tumours. Development of standardized research databases and well-designed comparative studies that control for selection and confounding biases are needed to further delineate these selection criteria.

## Electronic supplementary material

Supplementary file A: Search strategy (DOCX 13 kb)

Supplementary file B: Summary tables of study characteristics for Tuberculum Sellae meningioma and Olfactory Groove meningioma papers included in the meta-analysis (DOCX 54 kb)

Supplementary file C: Forrest plots for each tumour/approach/outcome combination (DOCX 1780 kb)

Supplementary file D: Funnel plots for each tumour/approach/outcome combination (DOCX 1428 kb)

Supplementary file E: Analysis of low of risk bias studies, compared with overall analysis (DOCX 23 kb)

## Abbreviations

TSM: Tuberculum sellae meningioma
OGM: Olfactory groove meningioma
eSKA: Endoscope-assisted supraorbital “keyhole” approach
EEA: Expanded endoscopic endonasal approach
mTCA: Microscopic transcranial approach
GTR: Gross total resection
CSF: Cerebrospinal fluid
WHO: World Health Organization
mNOS: Modified New-Castle Ottawa Scale
ICA: Internal carotid arteries
ACP: Anterior clinoid processes
LP: Lamina papyracea
CI: Confidence Interval
