# Supplementary material for: The endoscope-assisted supraorbital “keyhole” approach for anterior skull base meningiomas: an updated meta-analysis
Source: Acta Neurochir (Wien). 2020 Sep 5;163(3):661–76. doi: 10.1007/s00701-020-04544-x (PMC7474310; doi:10.1007/s00701-020-04544-x)
Supplement: Supplementary file 2 — Summary tables of study characteristics for Tuberculum Sellae meningioma and Olfactory Groove meningioma papers included in the meta-analysis (DOCX 54 kb) [file 701_2020_4544_MOESM2_ESM.docx]

Table 4: Study characteristics for Tuberculum Sellae Meningioma papers. NS: Not specified, DM: diameter, EEA: endoscopic transsphenoidal approach, mTCA: microscopic transcranial approach, eSKA: Supraorbital Keyhole Approach. SD: Standard deviation, mNOS: modified-New-Castle Ottawa Scale, which assesses study quality by examining sample selection and outcome reporting. *estimated mean based on median, number and range as per Hozo et al.

| Authors | TSM (N) | Approach | Mean Age (Range) | Meningioma WHO Grade 1 (N) | % Male | Meningioma Size (Range) | Mean Follow-Up in months (Range) | mNOS |
| --- | --- | --- | --- | --- | --- | --- | --- | --- |
| Ali et al. | 30 | mTCA | 48 (34-63) | 30 | 43 | NS | 18 (6 – 48) | 3 |
| Bander et al. | 32 | mTCA + EEA | 55 | NS | 52.4 | TCA 5cm^3^, EEA: 5.58cm^3^ | TCA: 36, EEA: 24 | 5 |
| Bernat et al. | 35 | mTCA + EEA | 59 (55-63) | NS | NS | NS | 41 | 5 |
| Bassiouni et al. | 62 | mTCA | 53 (29-81) | NS | 26 | NS | 72 (18 – 168) | 3 |
| Bohman et al. | 5 | EEA | 53 (24-77) | NS | 40 | Mean DM: 4.74 cm | 12.3* (2.2 – 17)) | 4 |
| Bowers et al. | 27 | mTCA + EEA | 54 (23-77) | NS | 18.5 | NS | NS | 3 |
| Cai et al. | 30 | eSKA | 51.5 (29-75). SD: 10.3 | 29 | 16.7 | Mean DM: 25.3mm | 58 | 6 |
| Catapano et al. | 7 | EEA* | NS | NS | NS | NS | 39* (6–72) | 4 |
| Ceylan et al. | 23 | EEA | 52.9 (23-77) | NS | 18.5 | Mean DM 2.55 cm | NS (2 – 70) | 3 |
| Chokyu et al. | 34 | mTCA | 55.7 (23-78) | 34 | 15 | Mean DM: 2.43 cm | 95.8 (15 – 194) | 3 |
| Chowdhury et al. | 6 | EEA | 39.5 (29-52) | NS | 33 | Mean DM: 3.5 cm | 7 | 4 |
| Cook et al. | 3 | EEA | 40.3 (32-55) | NS | 0 | NS | NS | 3 |
| Curey et al. | 20 | mTCA | 59.1, SD: 11.1 | 20 | 15 | Mean DM: 3.25, SD: 1.38 cm | 56.3, SD: 34 | 4 |
| De Divitiis et al. | 51 | mTCA + EEA | NS | NS | 20 | DM: 6: < 2cm, 33: 2-4cm, 5: >4 cm | NS (9 – 252) | 4 |
| Della puppa et al. | 23 | mTCA | NS | NS | 0 | NS | 41 (3 – 77) | 3 |
| Dzhindzhikhadze et al. | 15 | eSKA | 57.1 (48-75) | 14 | 33.7 | Mean voliume: 15.2 (11.3 - 27.1) | 15.5 | 4 |
| Elshazly et al. | 25 | EEA | 53.9 | 25 | 16 | Mean volume: 5.29 cm3 (0.5-28) | 21 (1-53) | 6 |
| Engelhardt et al. | 20 | mTCA | 50.5 (43.5-59.6) | 20 | 15 | Mean DM: 19.3mm | 67.2 | 6 |
| Fatemi et al. | 14 | EEA | 51 SD - 15 | NS | 28.6 | Mean volume: 25 cm3, SD: 8mm | 27 | 6 |
| Fatemi et al. | 9 | eSKA | 49 SD - 7 | NS | 33.3 | Mean DM: 33mm | 14 | 6 |
| Gadgil et al. | 5 | EEA | 51 (31-66) | 5 | 40 | Mean volume: 6.3 cm^3^ | 15 (3 – 27) | 4 |
| Ganna et al. | 24 | mTCA | 53.8 (33-80) | 24 | 17 | Mean DM: 2.63 cm | 52 (18 – 92) | 3 |
| Goel et al. | 85 | mTCA | NS | NS | NS | NS | 48 (6 – 108) | 4 |
| Hayashi et al. | 22 | EEA | 58.2 (32-87) | NS | 31.8 | Mean DM: 24.3mm* | NS | 4 |
| Hayhurst et al. | 7 | EEA | 46 (33-65) | 7 | 42.9 | NS | 38.6* | 5 |
| Igressa et al. | 4 | eSKA | 59* (38-80) | NS | NR | Mean volume: 0.05 cm3* | 56* | 3 |
| Jang et al. | 24 | mTCA | 49.5 (25-70) | NS | 21 | Mean DM: 2.06 cm | 21 (3 – 54) | 3 |
| Khan et al. | 20 | EEA | 56.5 (31-81) | 20 | 30 | Mean volume: 11.98 cm^3^ | NS | 3 |
| Kitano et al. | 28 | mTCA + EEA | 55 (42-76) | NS | 14 | Mean volume; 8.1 mm3 (0.7–31.4) | NS | 3 |
| Kong et al. | 84 | EEA | 54.2, SD: 13.6 | NS | 23.8 | Mean DML 2.4 cm | 28 | 5 |
| Kong et al. | 94 | mTCA | 53.7 | NS | 23.4 | Mean DM: 2.1 cm | 28 | 5 |
| Koutourousiou et al. | 70 | EEA | 57.3 (36-88) | 70 | 16 | Mean DM: 2.3 cm | 29 (1 – 98) | 3 |
| Kuga et al. | 7 | EEA | 54.43 (34-72) | NS | 0 | Mean volume: 2.71 cm3 | 19 | 6 |
| Kuga et al. | 13 | mTCA | 56.92 | NS | 15.4 | Mean volume: 3.98 cm3 | 36.5 | 6 |
| Landeiro et al. | 23 | mTCA | 56.2 ( 38-77) | NS | 35 | NS | 31.2 (6 – 124) | 3 |
| Leveque et al. | 18 | mTCA | 63.8 (31-88) | NS | NS | DM <4.0 cm: 11, >4.0 cm: 7 | 56.9, SD: 32.9 | 4 |
| Li et al. | 43 | mTCA | 53.8 (24-68) | NS | 28 | DM: <2 cm: 8, 2-4 cm: 22, >4 cm: 13 | 64.8 (24 – 120) | 3 |
| Li-hua et al. | 67 | mTCA | 48.7 (28-76) | NS | 42 | DM: <3 cm: 29, >3cm: 38 | 29.3 (6 – 4.85) | 4 |
| Linsler et al. | 6 | EEA | 63.6, SD: 12.4 | 4 | 0 | Mean volume: 2.1cm3, SD: 0.8cm | 15.3, SD: 5.9 | 5 |
| Linsler et al. | 16 | eSKA | 60.9, SD: 8.1 | 15 | 31.3 | Mean volume: 14.9 cm3 | 19.6 | 5 |
| Liu et al. | 19 | mTCA | NS | NS |  | NS | 14.9 (4 – 46) | 4 |
| Magill et al. | 44 | EEA | NS | NS | NS | NS | 45.7* | 5 |
| Magill et al. | 95 | mTCA | NS | NS | NS | NS | 45.7* | 5 |
| Mahmoud et al. | 58 | mTCA | 56 (13-80) | NS | 31 | Mean DM: 2.9 | 23 | 4 |
| Margalit et al. | 51 | mTCA | 57.1 (28 -83) | NS | 32 | Mean max DM 2.94 cm, SD: 1.07 | 42.1 (2 – 84) | 3 |
| Marx et al. | 7 | mTCA | 50 | 7 | 28.6 | Mean volume: 9.57 cm3 | 32* | 5 |
| Marx et al. | 8 | eSKA | 58.13 (49-69) | 8 | 12.5 | Mean volume: 12 cm3 | 32* | 5 |
| Mathiesen et al. | 29 | mTCA | 58.3 (30 -84) | 29 | 21 | Mean max DM: 2.39 cm | 72 (18 - 120) | 4 |
| Ming et al. | 23 | mTCA | 52.2 | 23 | 26.1 | Mean DM: 30.6mm | 39.5* | 5 |
| Mortazavi et al. | 5 | mTCA | 51.6 | 5 | 40 | Mean DM: 17.4mm | 62.4 | 5 |
| Nakamura et al. | 72 | mTCA | 54.3 ( 30 -86) | 71 | 24 | Mean DM: 2.5 cm | 63.4 (4 – 270) | 3 |
| Nanda et al. | 24 | mTCA | NS | NS | NS | DM: <3 cm: 3, 3-5 cm: 6, >5 cm: 21 | 18 months (Median only) | 4 |
| Ogawa et al. | 29 | EEA | 58.9 (43 -79) | 27 | 26 | NS | 35.8 (6 – 59) | 3 |
| Ottenhausen et al. | 36 | mTCA + EEA | TCA: 64.3, EEA: 57.2 | N | 36.1 | TCA: 28.5, EEA: 6.8 | 42.2 | 5 |
| Padhye et al. | 3 | EEA | 66 (65 - 66) | 3 | 0 | Mean volume 9.3 cm^3^ | 3 | 4 |
| Paiva-neto et al. | 9 | eSKA | 49.89 (33-62) | 9 | 0 | Mean volume: 14.89 cm3 | 31.5 | 5 |
| Palani et al. | 41 | mTCA | NR | NS | 37 | NS | NS (0.5 – 4) | 4 |
| Pamir et al. | 42 | mTCA | 53 (24-79) | 38 | 33 | NS (7.5-210 mm^3^) | 30 (3 - 192) | 3 |
| Park et al. | 21 | mTCA | 51 | NS | 14 | Mean volume: 12.4 cm^3^ | 75.9 (12 – 151) | 4 |
| Refaat et al. | 16 | mTCA | NS | NS | 19 | Mean DM: 2.5cm | 14 (8 – 18) | 3 |
| Romani et al. | 52 | mTCA | 59 (14-87) | 51 | 19 | Mean DM: 3.1 cm | 59^+^ (1 – 133) | 3 |
| Schick et al. | 53 | mTCA | 52.6 (27-78) | NS | 25 | Mean DM 2.6 cm | 29.9 (6 – 108) | 4 |
| Schroeder et al. | 3 | eSKA | 58.33 (55-61) | 3 | 0 | Mean volume: 12.4 cm3 | 47.5 | 6 |
| Seol et al. | 86 | mTCA | 49 (24-75) | NS | 23 | Mean Dm: 2.41 | 39 (6 – 146) | 3 |
| Song et al. | 44 | EEA | 52.7 (26-76) | 40 | 13.6 | Mean volume: 5.8 cm3 | 27 | 5 |
| Song et al. | 40 | mTCA | 54.4 | 39 | 15 | Mean volume: 5.8 cm3 | 43.5 | 5 |
| Telera et al. | 14 | eSKA | 56.93 (39-72) | NS | 14.3 | Mean volume: 9.75 cm3 | 48* | 6 |
| Terasaka et al. | 9 | mTCA | 64 (57-83) | 9 | 11 | NS | 25.2 (0.5 - 5.92) | 4 |
| Voznyak et al. | 26 | mTCA | 48.7 | 16 | 23.1 | Mean DM: 31.8 mm | NS (3-108) | 4 |
| Wang et al. | 12 | EEA | 56.7 (40 -67) | 12 | 33 | Mean DM: 3.03 cm | 25.2 (6 – 60) | 3 |
| Wilk et al. | 18 | mTCA | 50.5 (30 - 73) | 18 | 16.7 | Mean volume: 6.92 cm3 | 20.6 (6 – 39) | 6 |
| Xiao et al. | 4 | eSKA | 52 (33-62) | NS | 25 | NS | NS | 2 |
| Zhou et al. | 56 | mTCA | 42.5 (21-69) | NS | 46 | DM: <3 cm: 24, 3-5 cm: 26 > 5m: 6 | 27.47 (1 – 36) | 4 |
| Zoli et al. | 35 | EEA | NS | 21 | NS | NS | 58 | 4 |

Table 5: Study characteristics of OGM papers. NS: Not specified, DM: diameter, EEA: endoscopic transsphenoidal approach, mTCA: microscopic transcranial approach, eSKA: Supraorbital Keyhole Approach. SD: Standard deviation, mNOS: modified-New-Castle Ottawa Scale, which assesses study quality by examining sample selection and outcome reporting. *estimated mean based on median, number and range as per Hozo et al.

| Authors | OGM (N) | Approach | Mean Age (Range) | Meningioma WHO Grade 1 (N) | % Male | Meningioma Size (Range) | Mean Follow-Up in months (Range) | mNOS |
| --- | --- | --- | --- | --- | --- | --- | --- | --- |
| Aguiar et al. | 21 | mTCA | 50 (21-76) | NS | 29 | Mean DM: 4.3, SD: 1.1 cm | 50 (3 – 120) | 3 |
| Al-meida et al. | 10 | EEA | 53.1 | NS | 30 | Mean volume: 35.7 cm3 | 54* (3 – 115) | 4 |
| Al-meida et al. | 10 | mTCA | 49.7 | NS | 20 | Mean volume: 36.2 cm3 | 31.5* (2 - 89) | 4 |
| Banu et al. | 6 | EEA | 67.3 (48–77) | NS | 0 | Mean volume:19.6 cm3 (7–49.6) | 18.5* (1 – 80) | 5 |
| Banu et al. | 7 | eSKA | 59.3 (41–73) | NS | 42.9 | Mean volume: 33.5 cm3 (7.3–61.4) | 18.5* 1 – 80) | 5 |
| Barzaghi et al. | 21 | mTCA | 54.1 (28-69) | 19 | 42.9 | Mean DM: 45.9 mm (25-70)* | 87 (36-176), SD: 7 | 5 |
| Bassiouni et al. | 62 | mTCA | 51 (NS) | 61 | 27 | Mean DM: 5.2 cm | 67.2 (12 – 156) | 4 |
| Bernat et al. | 19 | EEA + mTCA | 59 | EEA: NS, TCA: 30.8 | NS | NS | EEA: 38, TCA: 44 | 5 |
| Bitter et al. | 61 | mTCA | 60 (NS) | 54 | 34 | DM: <2 cm: 5%, 2 - 4 cm: 6.5%, >4 cm: 88.5% | 122 (8 – 239) | 3 |
| Ciurea et al. | 59 | mTCA | 52.9 (20-76) | 56 | 41 | DM: 2-4cm: 16, 4-6: 32, >6: 11 | 84 (9 – 144) | 3 |
| Colli et al. | 17 | mTCA | 53.12 (19-76) | 17 | 6 | NS | 51 (1 – 209) | 4 |
| De divitiis et al. | 4 | EEA | 49.25 (35-65) | 4 | 25 | Mean DM: 4.0 cm (SD: NR) | 9.7 (9 – 12) | 3 |
| Della puppa et al. | 20 | mTCA | NS | NS | NS | DM: < 3.5 CM | 41 (3 – 77) | 4 |
| Eroglu et al. | 14 | eSKA | 47.42 SD - 19.6 | NS | 57.1 | NS | 33.7, SD:18.35 | 4 |
| Farooq et al. | 19 | mTCA | 51 (35-67) | NS | 5.26 | Mean DM: 6cm | 60 | 5 |
| Fountas et al. | 78 | mTCA | 63.3 | 76 | 39.7 | NS | 67.2 (24-180) | 5 |
| Goel et al. | 129 | mTCA | 46 (18-75) | NS | 15.5 | NS | 82 (6-348) | 4 |
| Guduk et al. | 63 | mTCA | 58 (33-80) | 59 | 31.1 | Mean DM: 4.9 cm (1.5-9)* | 64 (1-239)* | 5 |
| Hayhurst et al. | 9 | EEA | 50.2 (30 - 76) | 9 | 11.1 | NS | 38.6 (12 – 60)* | 5 |
| Igressa et al. | 17 | eSKA | 59 (38-80)* | NS | 30 | Mean DM: 0.05 | 56* | 3 |
| Jang et al. | 40 | mTCA | 59.1 (33-74) | 32 | 58 | Mean DM: 4.59 cm (SD: NS) | 59 (3 – 184) | 3 |
| Karsy et al. | 49 | mTCA | 52.3 | 46 | 24.5 | 12.4 | 24.3 | 5 |
| Khan et al. | 11 | EEA | NS | 10 | NS | NS | NS | 4 |
| Koutourousiou et al. | 45 | EEA | 57.1 (27-88) | 44 | 36 | Mean DM: 4.41 cm (SD: NR) | 32.5 (3 – 115) | 3 |
| Leveque et al. | 34 | mTCA | NS | NS | NS | NS | 57 (6 – 120) | 5 |
| Liu et al. | 5 | EEA | 51.1 (28-58) | NS | 20.0 | Mean volume: 33.3 cm3 (15.69–60.77) | 14.5 (1–76) | 5 |
| Liu et al. | 15 | mTCA | 52.13 (34–66) | NS | 33.3 | Mean volume: 92 cm3 (4.35–300.06) | 14.5 (1–76) | 5 |
| Ming et al. | 29 | mTCA | 54.9 (38 - 73) | 26 | 37.9 | Mean DM: 43.1 mm (23 - 69)* | 39.5* (16 - 64) | 5 |
| Mukherjee et al. | 33 | mTCA | 41 (4-89) | 21 | 33 | NS | 38 (6 - 62) | 4 |
| Nakamura et al. | 82 | mTCA | 57.8 (33-91) | NS | 23 | Mean DM: 4.5 cm | 64 (4 – 270) | 3 |
| Nanda et al. | 57 | mTCA | NS | NS | 40 | Mean DM: 4.41 cm | 14.12 (12 – 16) | 3 |
| Ottenhausen et al. | 12 | eSKA + EEA | SKA: 65.2, EEA: 78.5 | NS | SKA: 10, EEA: 0 | SKA: 21.1cm^3^, EEA: 10.8cm^3^ | 42.2 | 5 |
| Padhye et al. | 8 | EEA | 52 (28-74) | 8 | 25 | Mean volume: 41.3 cm^3^ | 23.6 (3 – 60) | 3 |
| Paiva-neto et al. | 12 | eSKA | 56 (48-68) | 12 | 16.7 | Mean volume: 44.8 cm3 (13.5 - 135) | 31.5 (6 – 66) | 5 |
| Pallini et al. | 113 | mTCA | 57 (17-82) | NS | 35 | Mean DM: 5.4 cm | 126* (2– 324) | 3 |
| Patel et al. | 50 | mTCA | 62.1 (23-84) | 50 | 26 | Mean volume: 48.8 cm3 | 59.3 (17-157) | 6 |
| Pepper et al. | 19 | mTCA | 51 (15-68) | 15 | 53 | NS | 41 | 3 |
| Refaat et al. | 14 | mTCA | 50.8 (35-67) | NS | 21 | Mean DM: 5.8 cm | 14 (9 – 18) | 3 |
| Reisch et al. | 21 | eSKA | NS | NS | NS | DM: 54.8% (2.5-4.4cm), 19.3% (>4.5cm) | 120 | 3 |
| Romani et al. | 66 | mTCA | 57 (38-85) | 58 | 47 | Mean DM: 4.7 cm | 45* (1 – 133) | 4 |
| Schroeder et al. | 5 | eSKA | 60.6 (42-78) | 5 | 60 | Mean volume: 28.62 cm3 (3.58 - 60.19) | 73.2 (55-91) | 6 |
| Slavik et al. | 29 | mTCA | 54 (36-68) | NS | 41 | NS | NS | 3 |
| Spektor et al. | 80 | mTCA | 55 (16-85) | 78 | 28 | Mean DM: 4.6 cm (SD: NR) | 70.8 (6 – 164) | 3 |
| Telera et al. | 5 | eSKA | 62 (43-81) | NS | 60 | Mean volume: 13.8 cm3 | 48* | 6 |
| Tuna et al. | 25 | mTCA | NS | NS | NS | NS | 58.4 (14 – 112) | 4 |
| Xiao et al. | 5 | eSKA | 54.6 (42-62) | NS | 60 | NS | NS | 2 |
