# Supplementary material for: The endoscope-assisted supraorbital “keyhole” approach for anterior skull base meningiomas: an updated meta-analysis
Source: Acta Neurochir (Wien). 2020 Sep 5;163(3):661–76. doi: 10.1007/s00701-020-04544-x (PMC7474310; doi:10.1007/s00701-020-04544-x)
Supplement: Supplementary file 3 — Forrest plots for each tumour/approach/outcome combination (DOCX 1780 kb) [file 701_2020_4544_MOESM3_ESM.docx]

**Appendix B: Forrest plots for each tumour/approach/outcome combination**

| **1) Gross total resection** | |
| --- | --- |
| **Olfactory Groove Meningioma** | **Tuberculum Sellae Meningioma** |
| 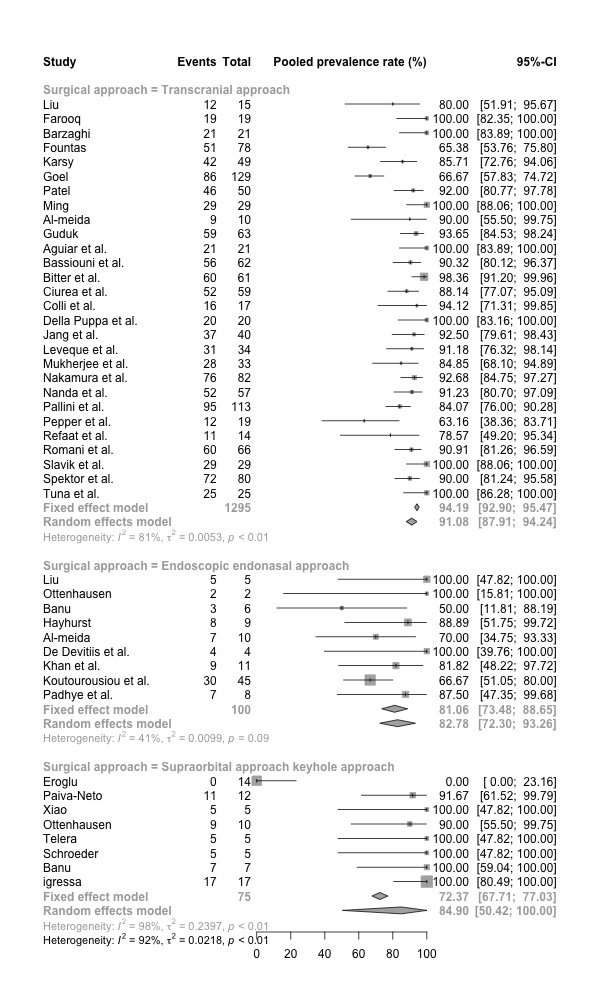 | 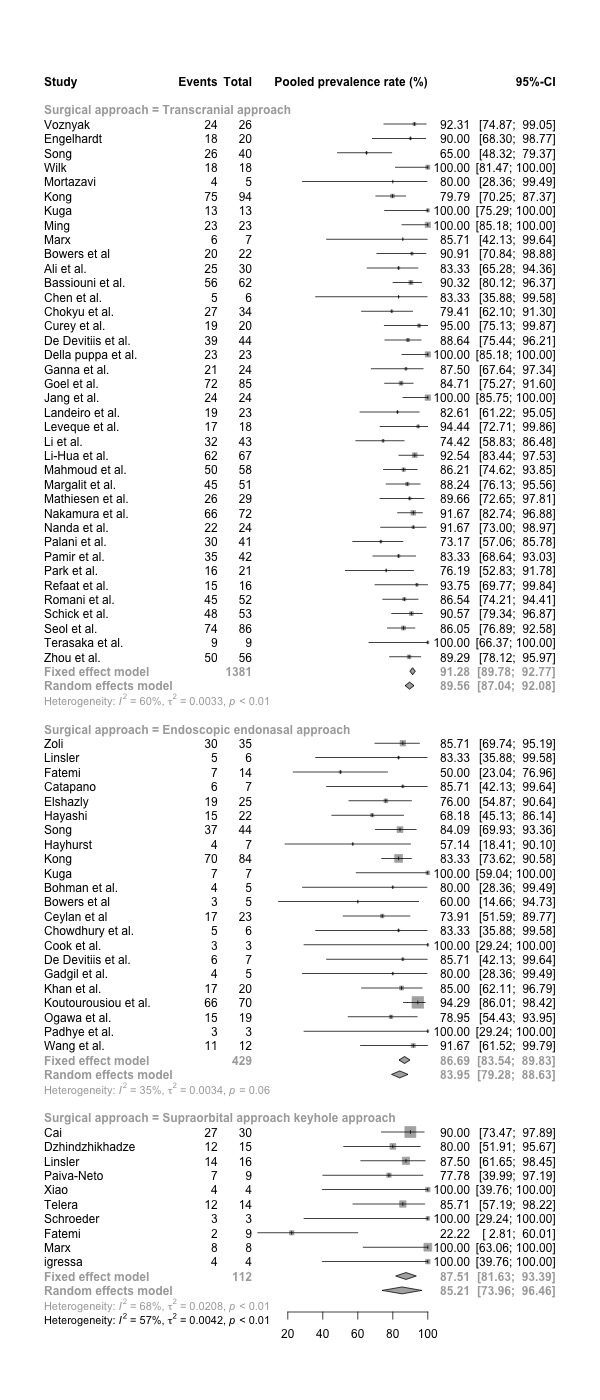 |

| **2) Visual Improvement** | |
| --- | --- |
| **Olfactory Groove Meningioma** | **Tuberculum Sellae Meningioma** |
| 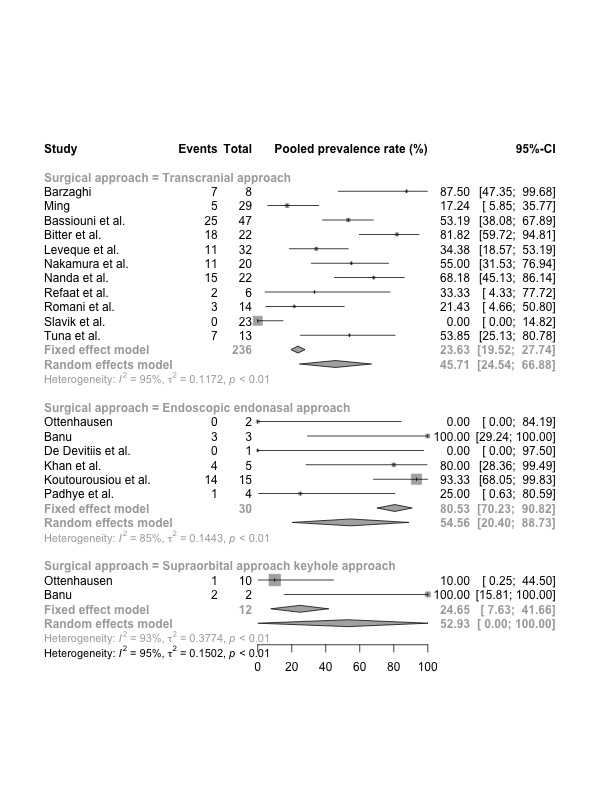 | 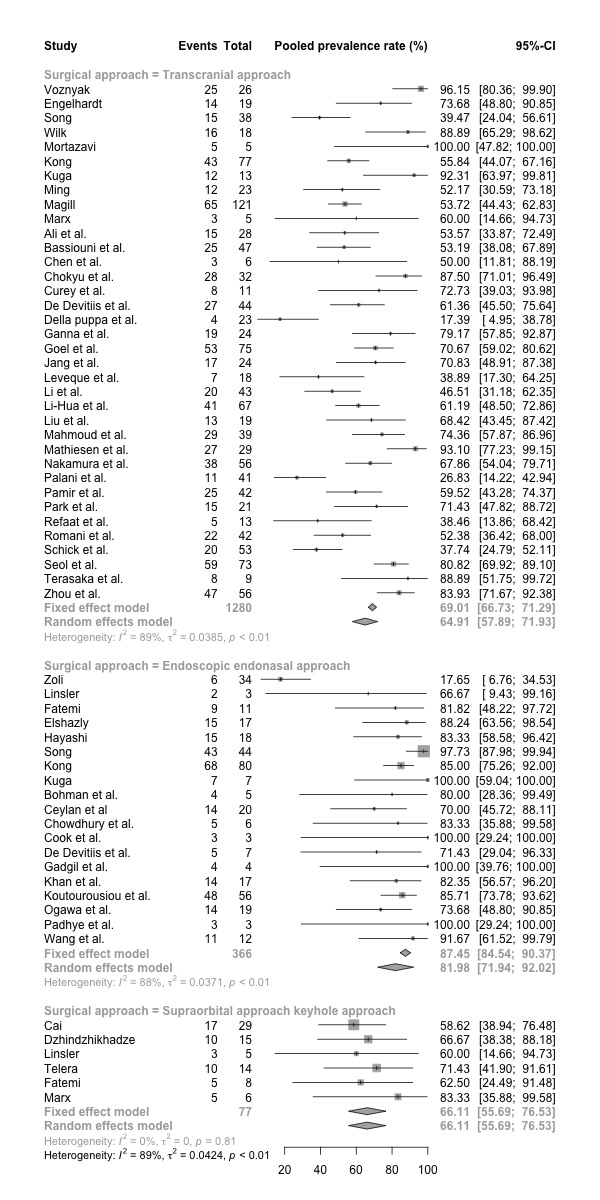 |

| **3) Post-operative cerebrospinal fluid (CSF) leak** | |
| --- | --- |
| **Olfactory Groove Meningioma** | **Tuberculum Sellae Meningioma** |
| 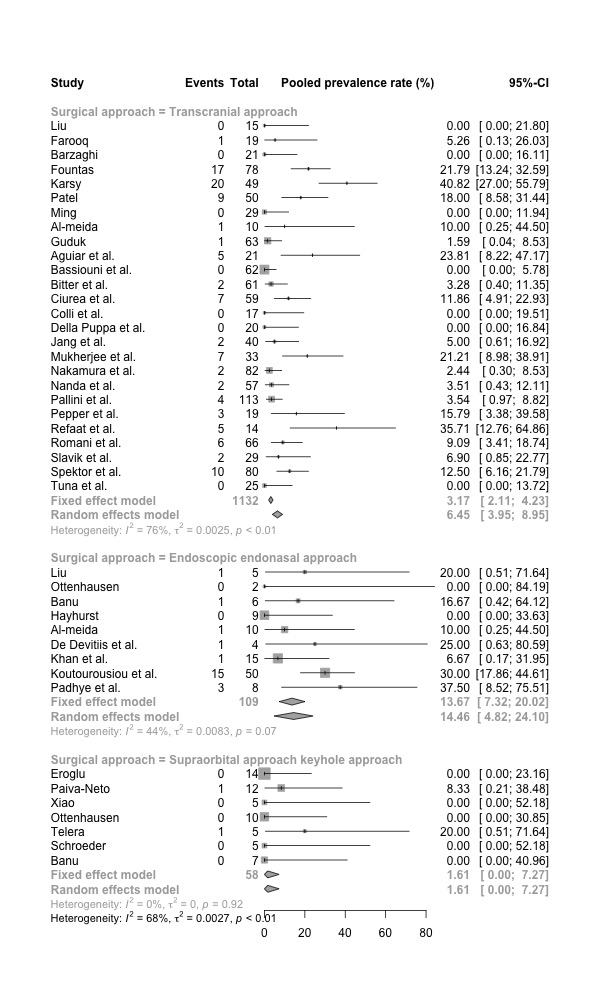 | 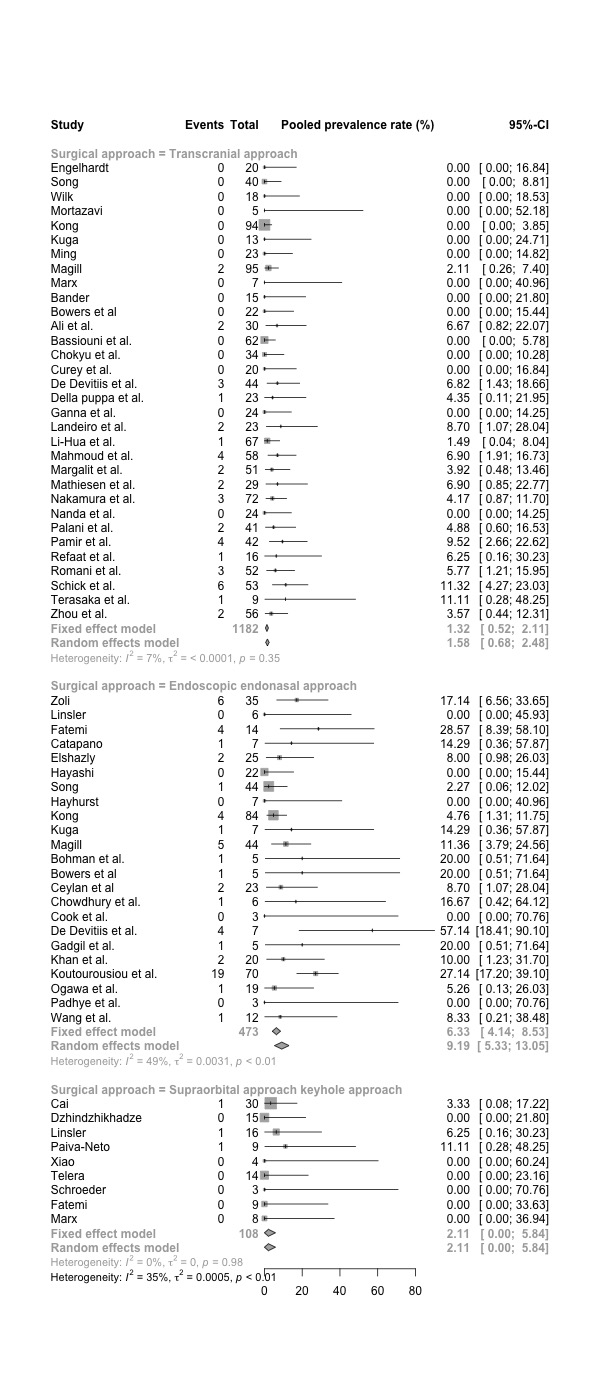 |

| **4) Intra-operative arterial injury** | |
| --- | --- |
| **Olfactory Groove Meningioma** | **Tuberculum Sellae Meningioma** |
| 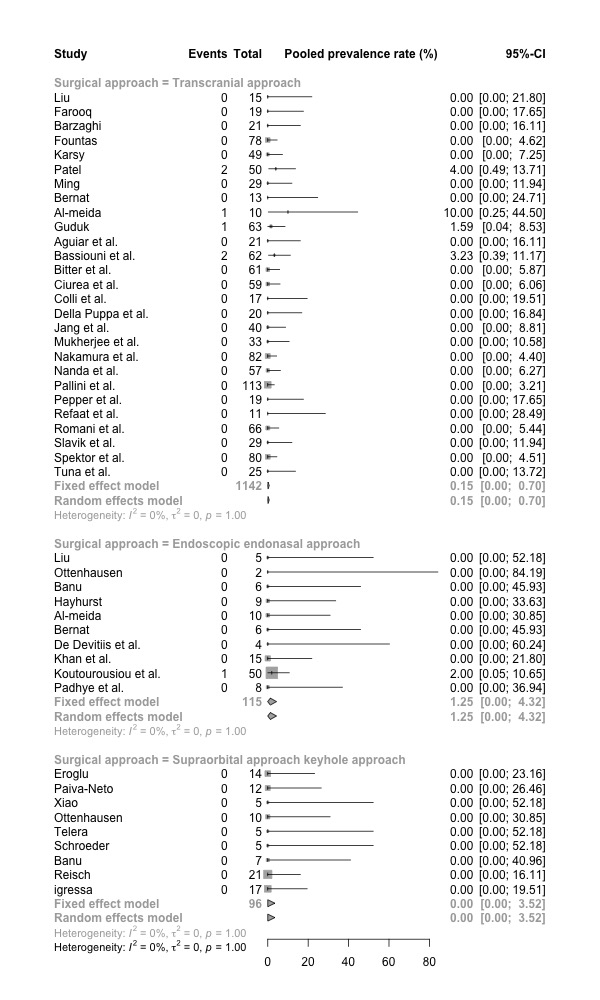 | 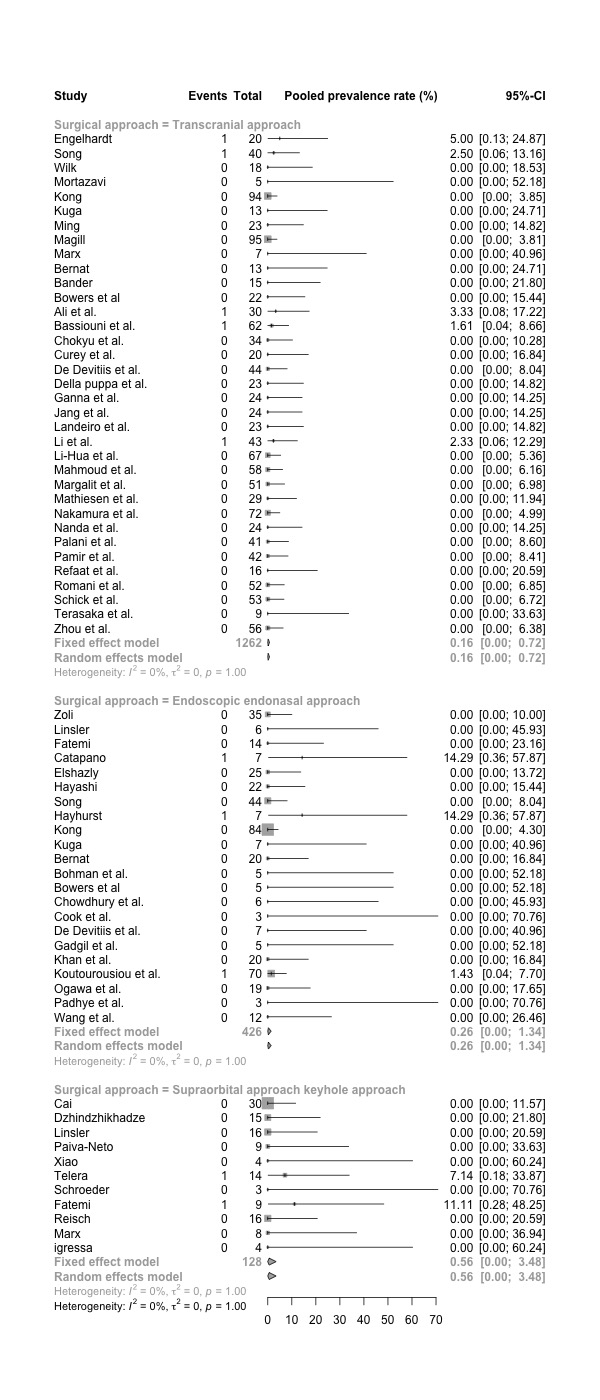 |

| **5) 30-day mortality** | |
| --- | --- |
| **Olfactory Groove Meningioma** | **Tuberculum Sellae Meningioma** |
| 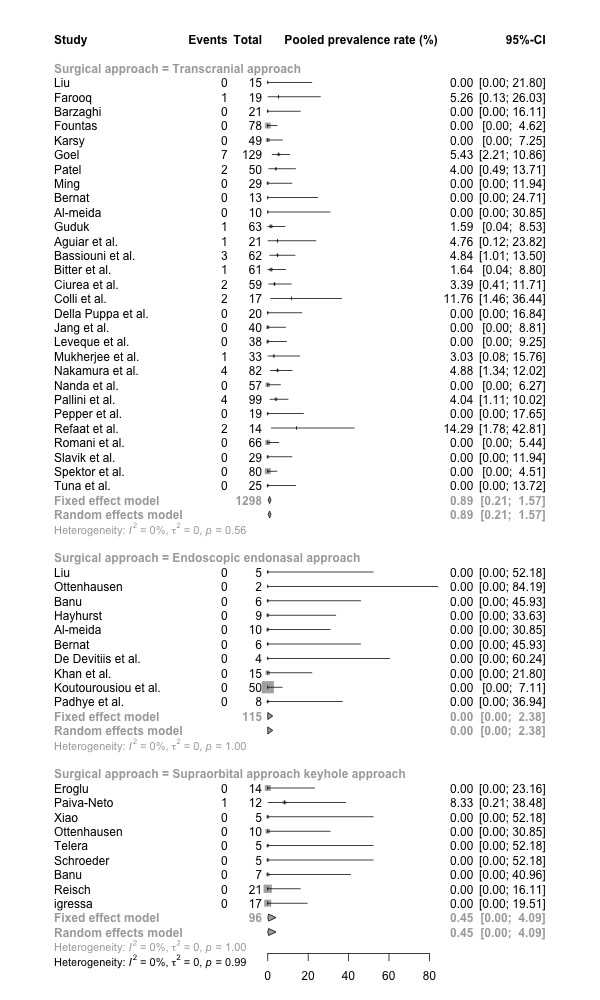 | 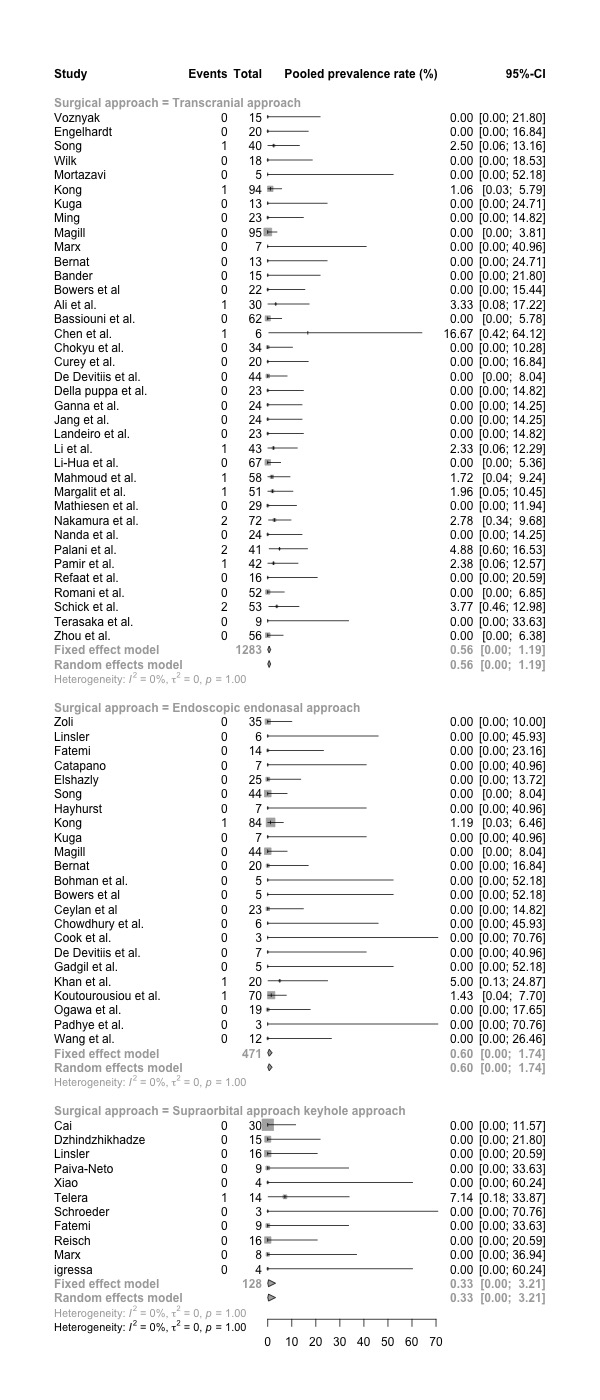 |
