# Supplementary material for: The endoscope-assisted supraorbital “keyhole” approach for anterior skull base meningiomas: an updated meta-analysis
Source: Acta Neurochir (Wien). 2020 Sep 5;163(3):661–76. doi: 10.1007/s00701-020-04544-x (PMC7474310; doi:10.1007/s00701-020-04544-x)
Supplement: Supplementary file 4 — Funnel plots for each tumour/approach/outcome combination (DOCX 1428 kb) [file 701_2020_4544_MOESM4_ESM.docx]

**Appendix D: Funnel plots for each tumour/approach/outcome combination**

**1) Gross total resection:**

| **Tuberculum Sellae Meningioma** | |
| --- | --- |
| Raw funnel plot | Trim & fill funnel plot |
| Expanded endoscopic endonasal approach | |
| 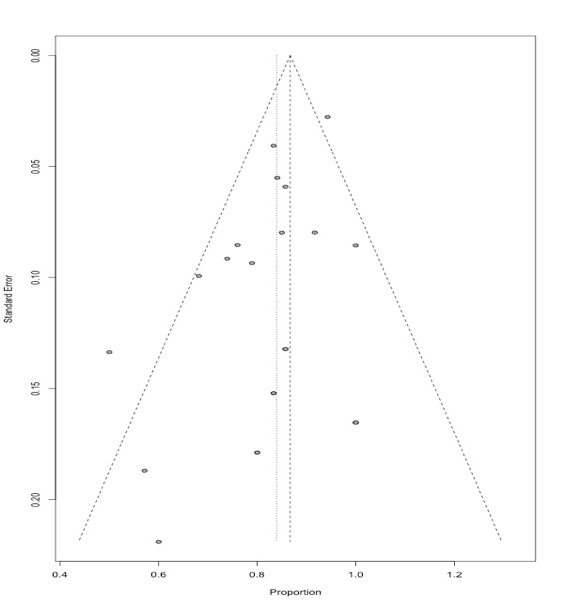 | 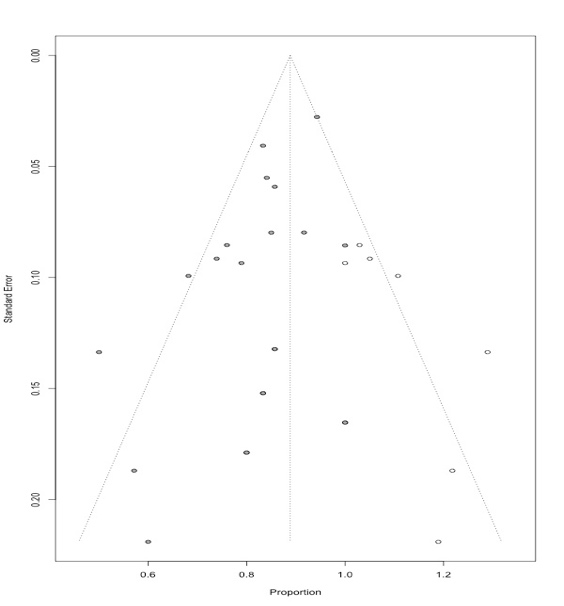 |
| Endoscope-assisted supraorbital keyhole approach | |
| 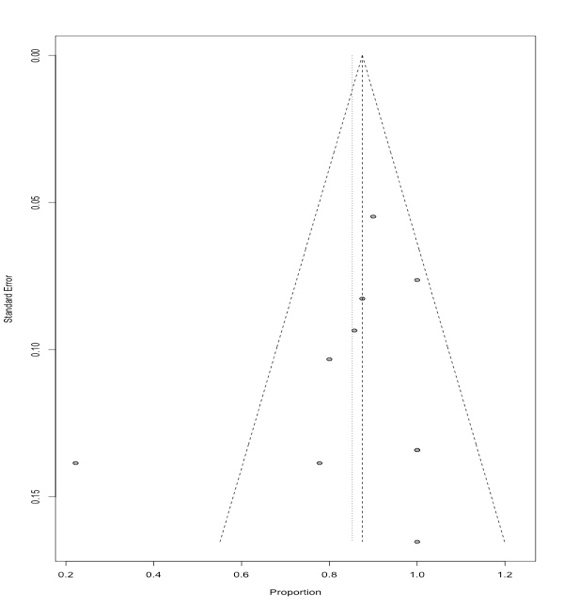 | 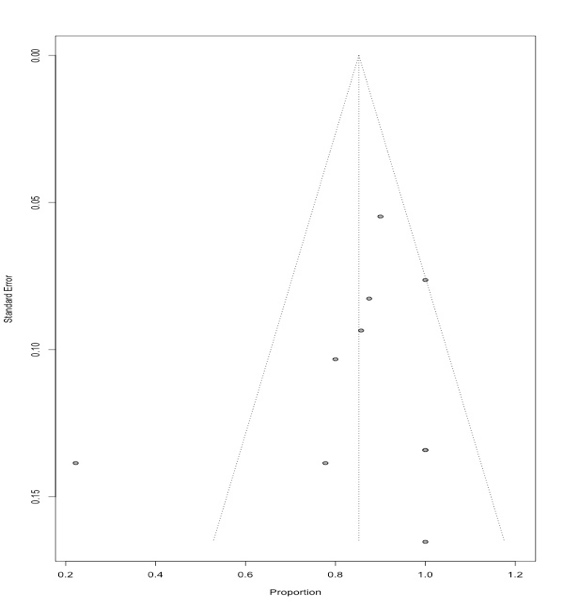 |
| Microscopic transcranial approach | |
| 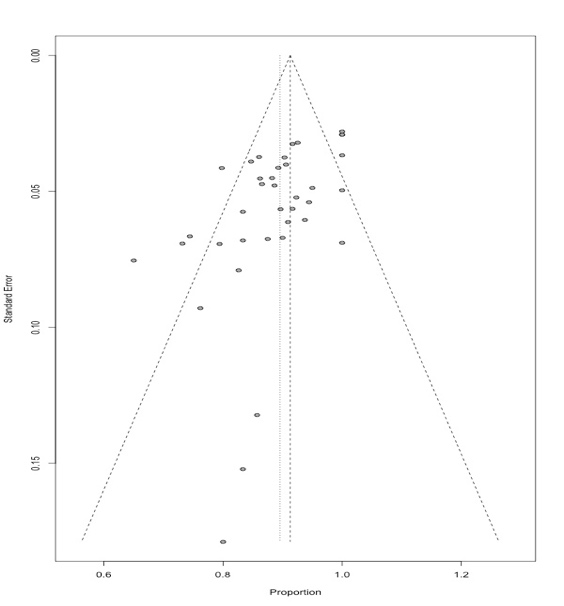 | 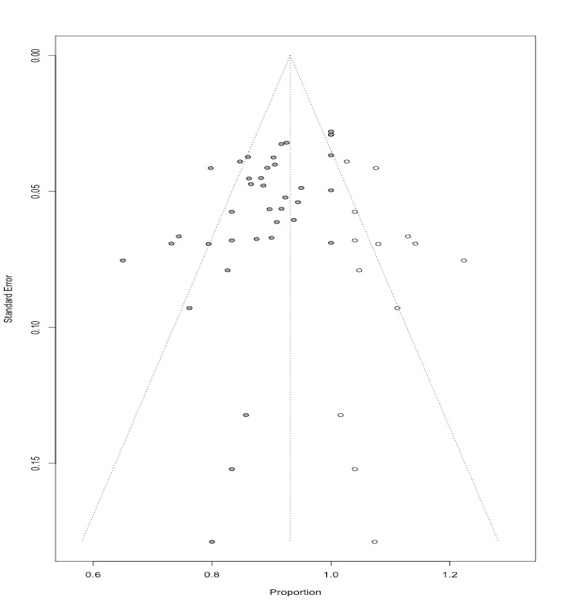 |
| **Olfactory Groove Meningioma** | |
| Raw funnel plot | Trim & fill funnel plot |
| Expanded endoscopic endonasal approach | |
| 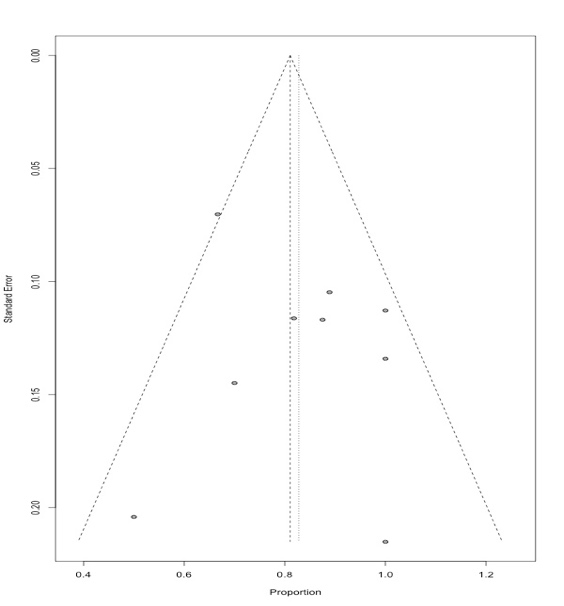 | 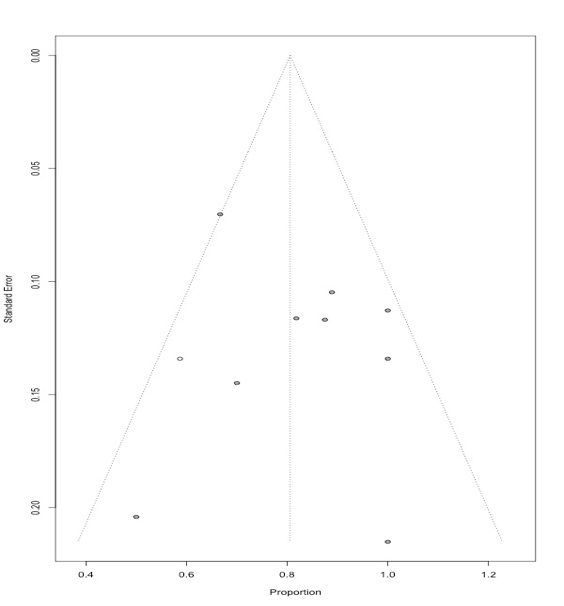 |
| Endoscope-assisted supraorbital keyhole approach | |
| 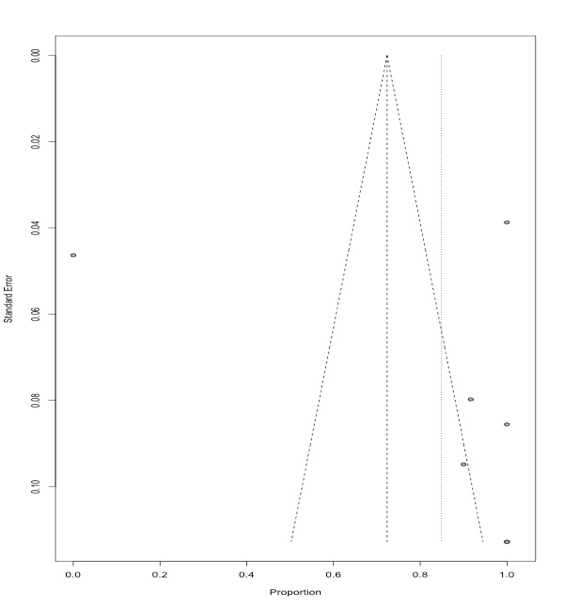 | 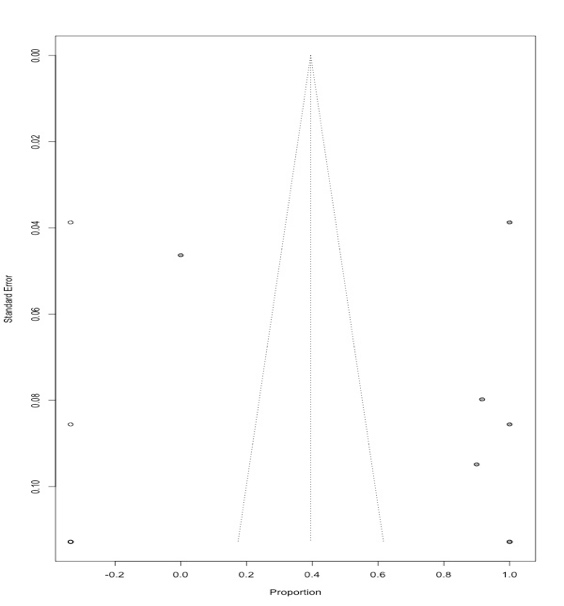 |
| Microscopic transcranial approach | |
| 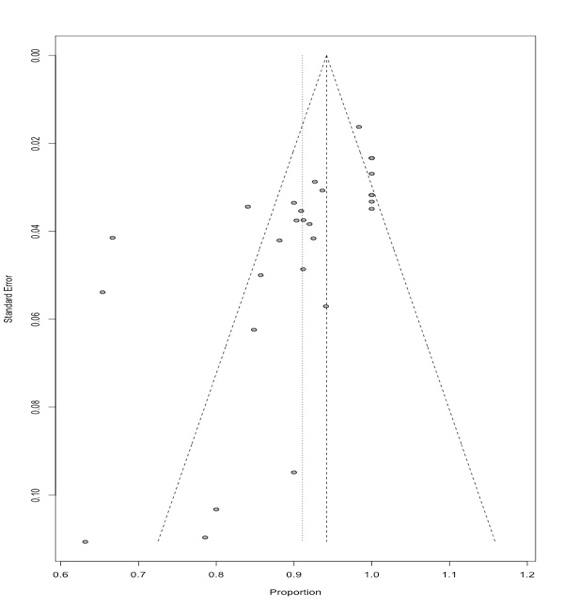 | 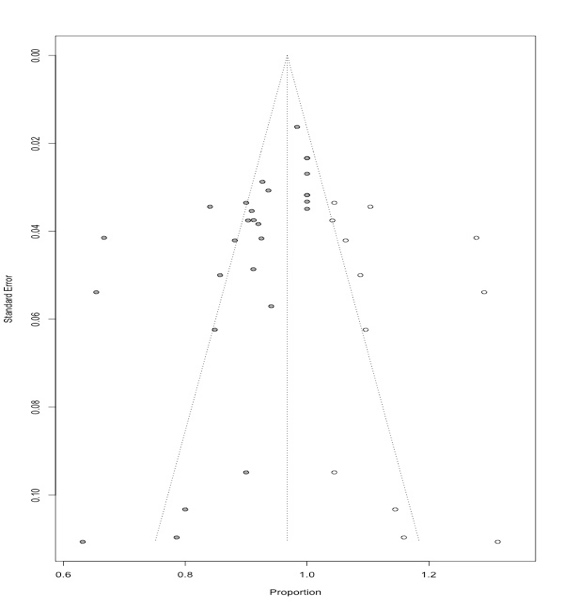 |

2) **Visual Improvement:**

| **Tuberculum Sellae Meningioma** | |
| --- | --- |
| Raw funnel plot | Trim & fill funnel plot |
| Expanded endoscopic endonasal approach | |
| 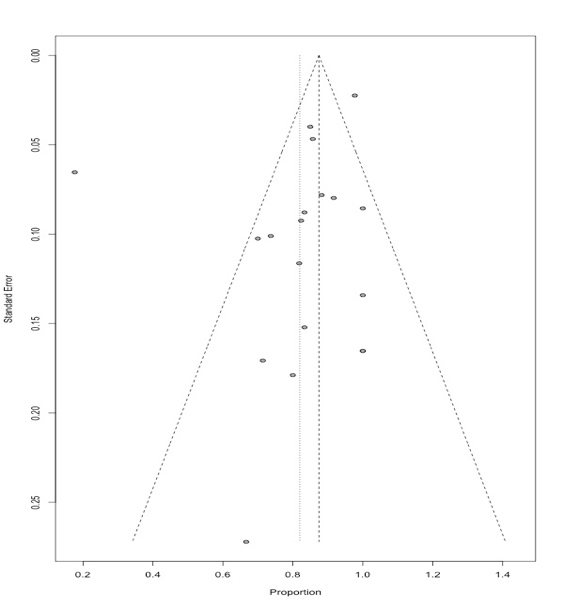 | 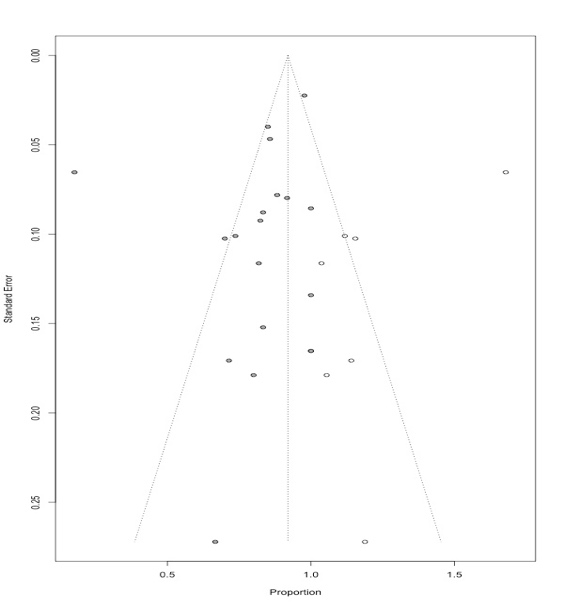 |
| Endoscope-assisted supraorbital keyhole approach | |
| Not available | Not available |
| Microscopic transcranial approach | |
| 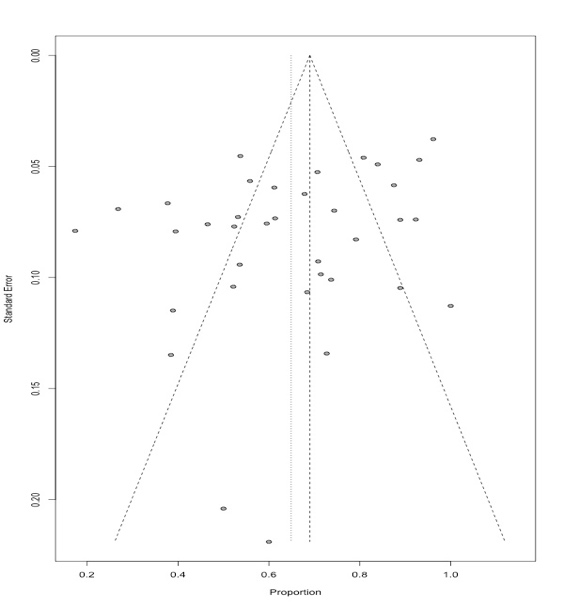 | 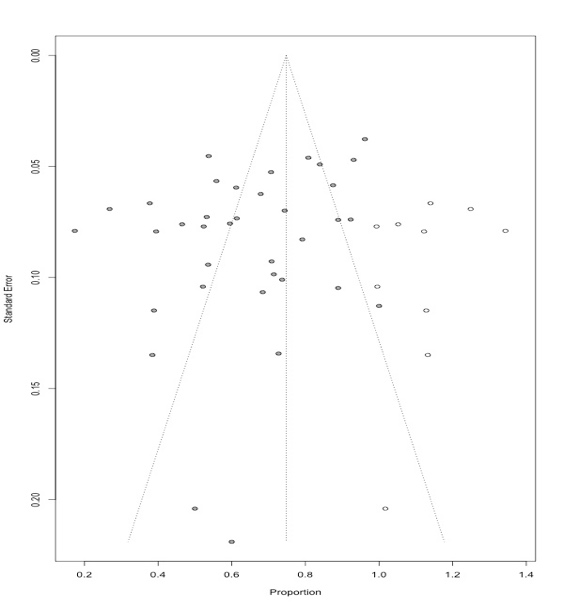 |

| **Olfactory Groove Meningioma** | |
| --- | --- |
| Raw funnel plot | Trim & fill funnel plot |
| Expanded endoscopic endonasal approach | |
| 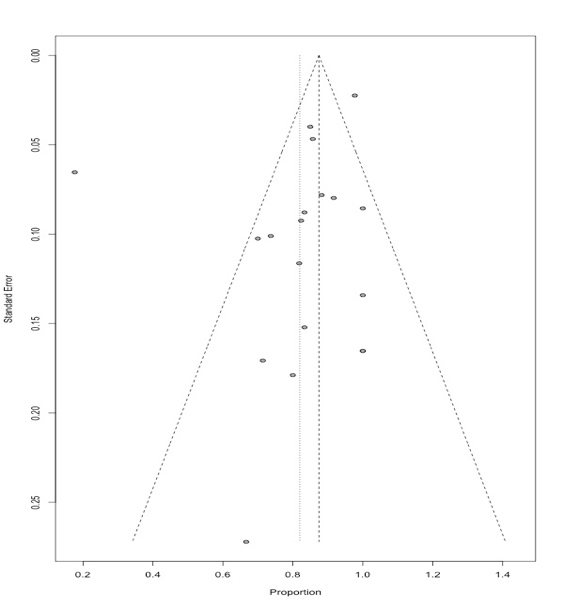 | 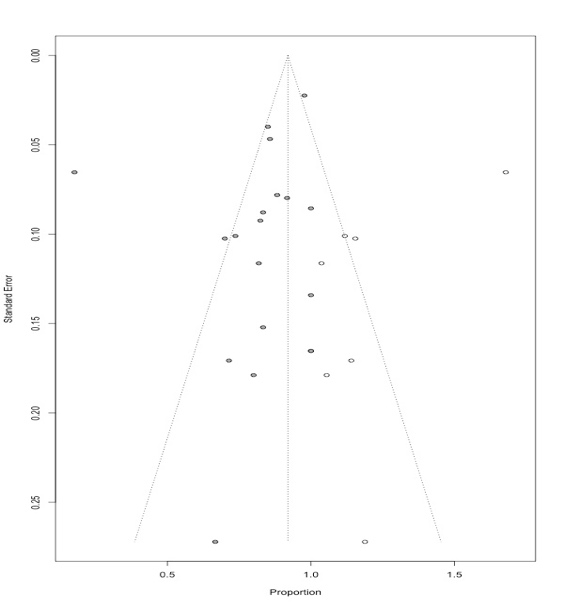 |
| Endoscope-assisted supraorbital keyhole approach | |
| Not available | Not available |
| Microscopic transcranial approach | |
| 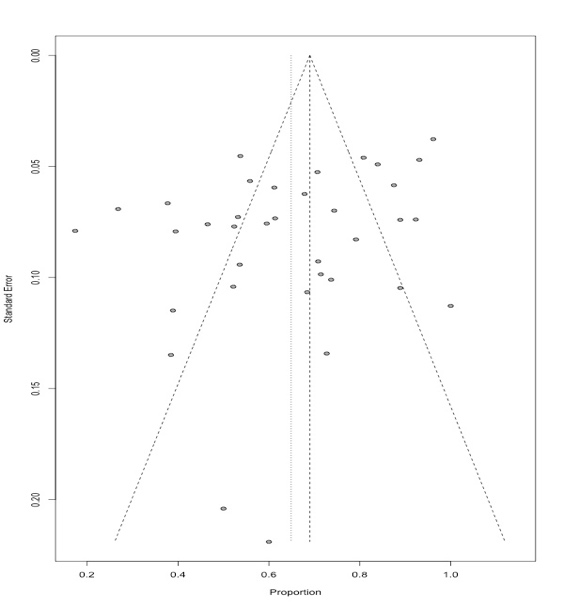 | 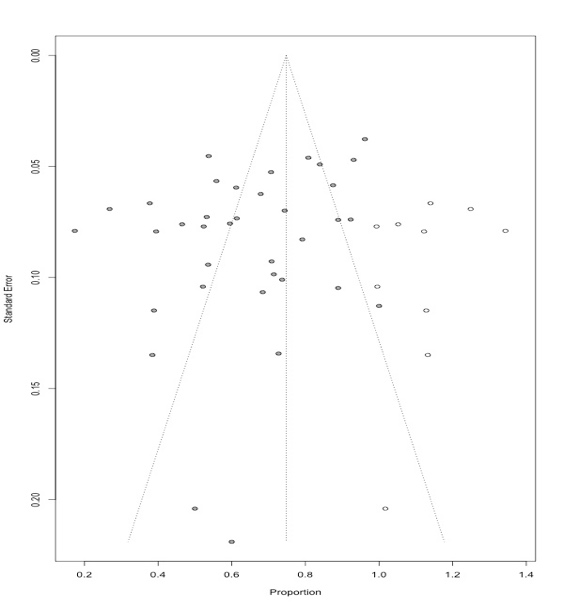 |

**3) Post-operative cerebrospinal fluid (CSF) leak**

| **Tuberculum Sellae Meningioma** | |
| --- | --- |
| Raw funnel plot | Trim & fill funnel plot |
| Expanded endoscopic endonasal approach | |
| 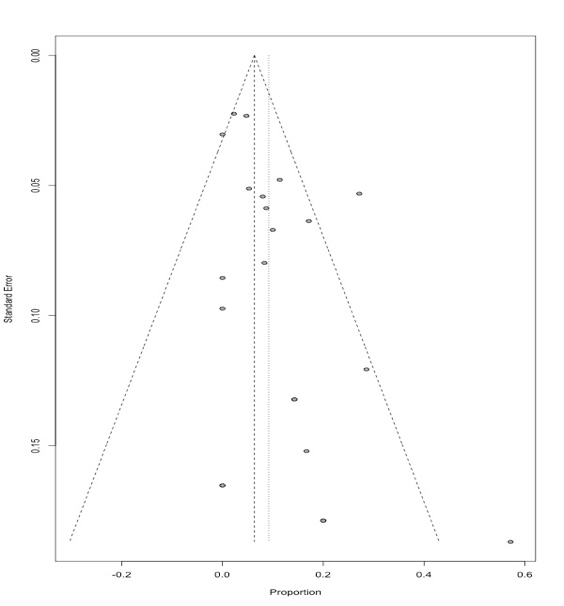 | 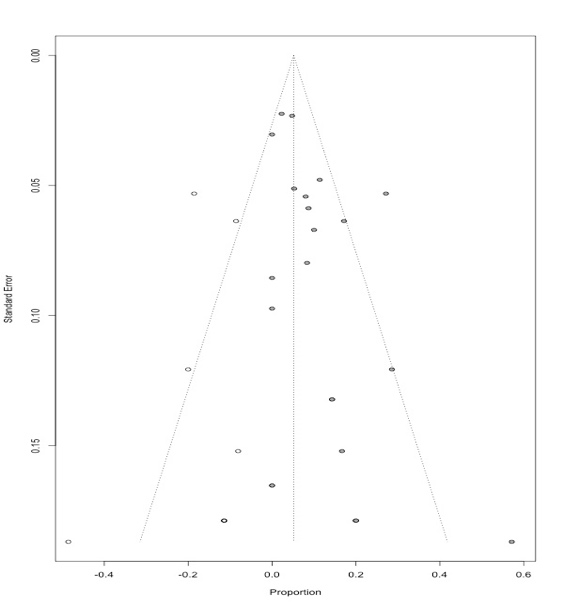 |
| Endoscope-assisted supraorbital keyhole approach | |
| 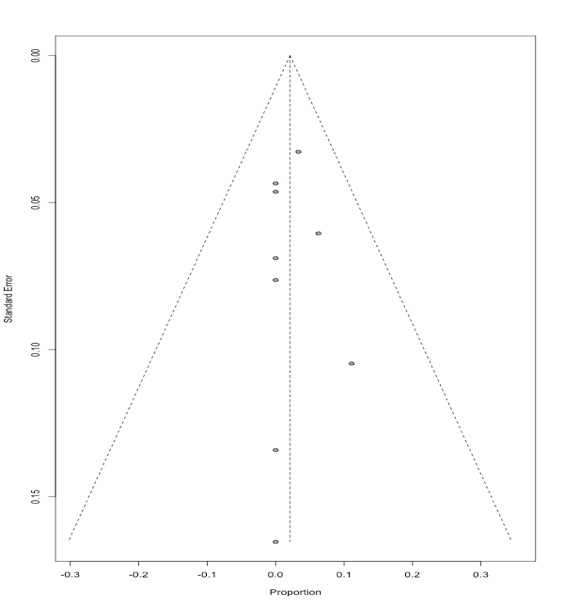 | 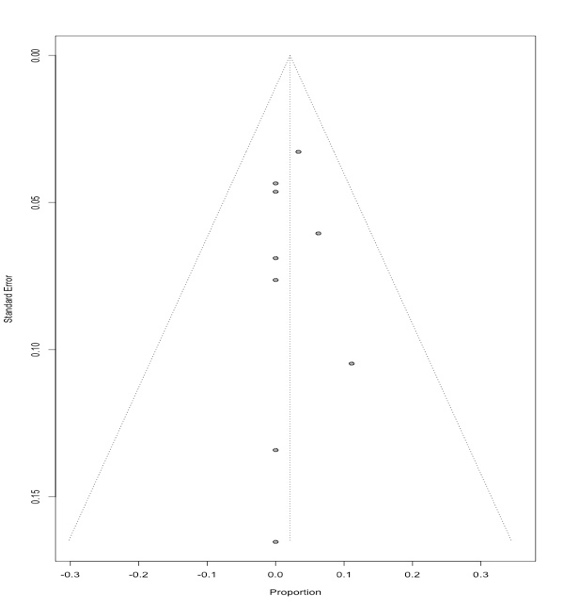 |
| Microscopic transcranial approach | |
| 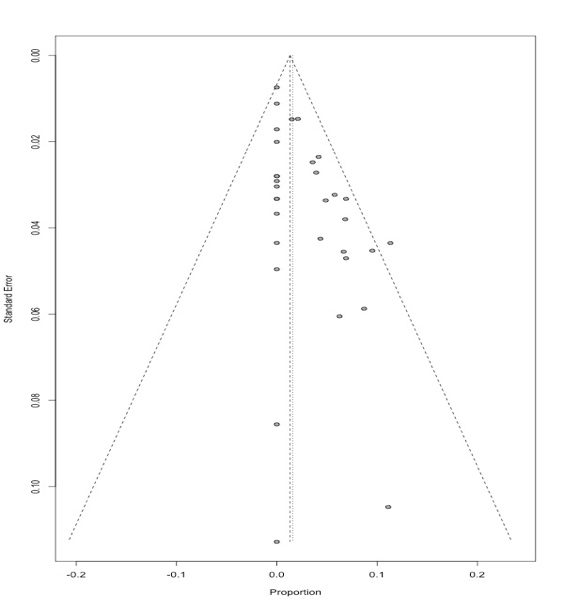 | 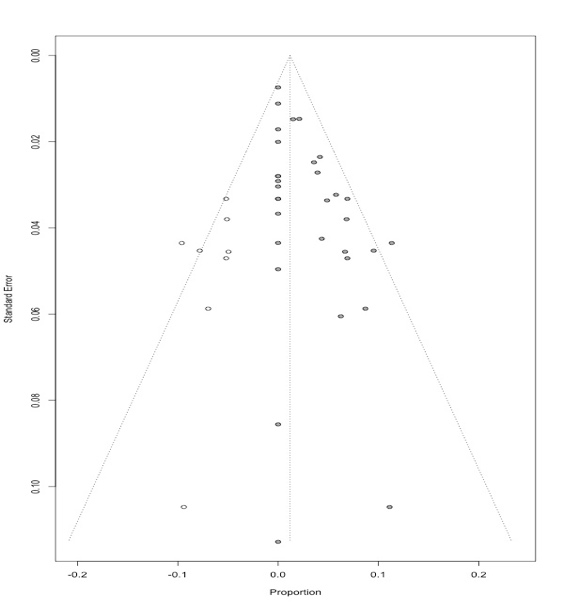 |

| **Olfactory Groove Meningioma** | |
| --- | --- |
| Raw funnel plot | Trim & fill funnel plot |
| Expanded endoscopic endonasal approach | |
| 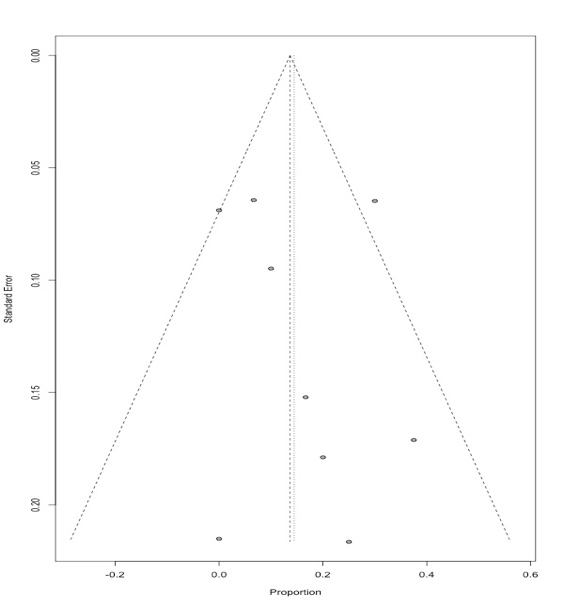 | 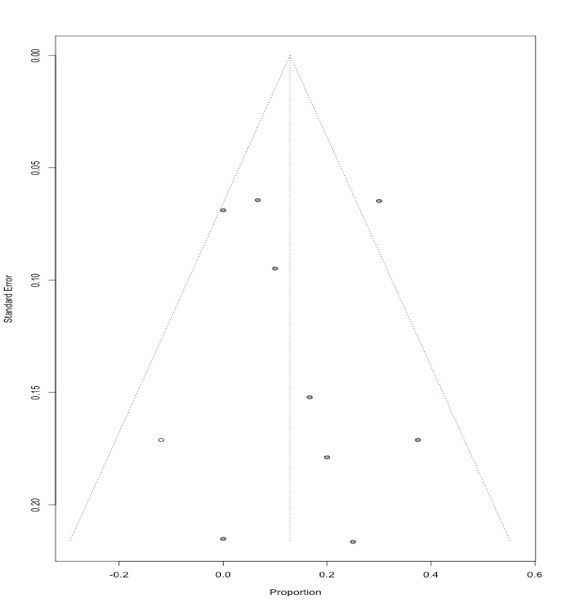 |
| Endoscope-assisted supraorbital keyhole approach | |
| Not available | Not available |
| Microscopic transcranial approach | |
| 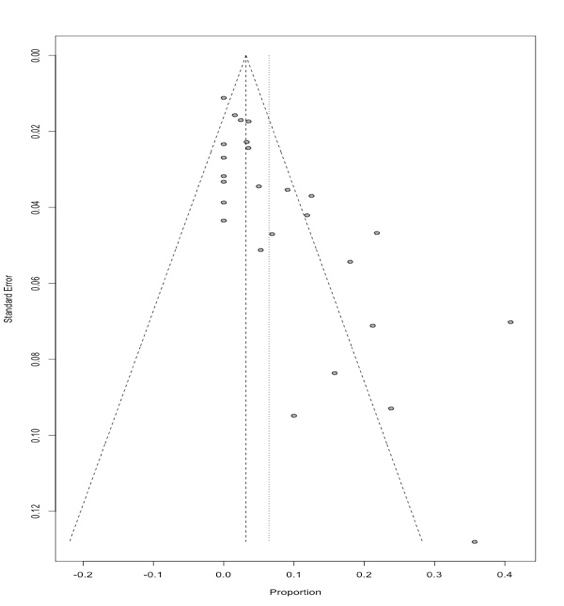 | 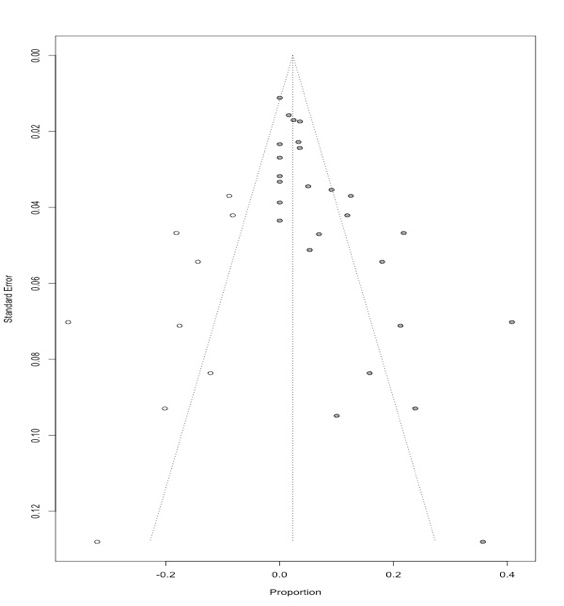 |

## 4) Intraoperative arterial injury:

| **Tuberculum Sellae Meningioma** | |
| --- | --- |
| Raw funnel plot | Trim & fill funnel plot |
| Expanded endoscopic endonasal approach | |
| 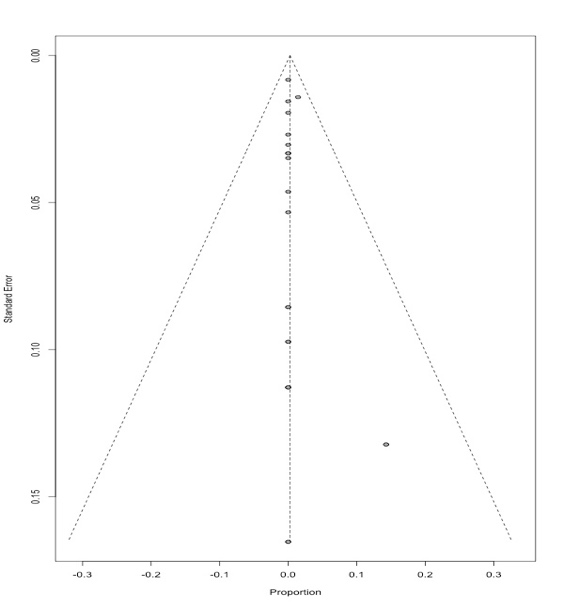 | 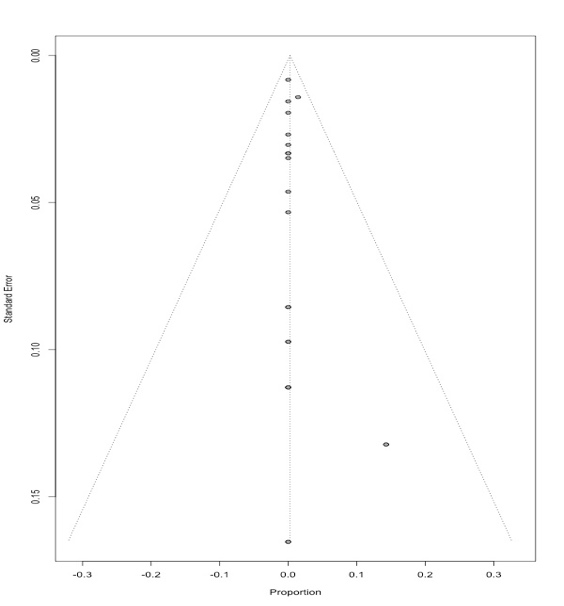 |
| Endoscope-assisted supraorbital keyhole approach | |
| 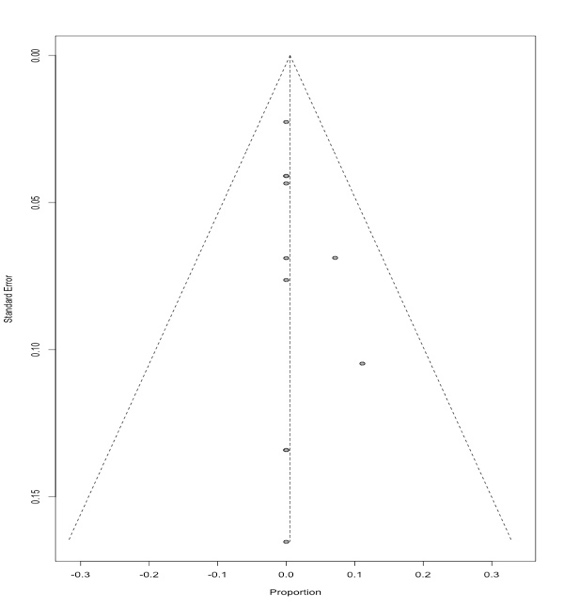 | 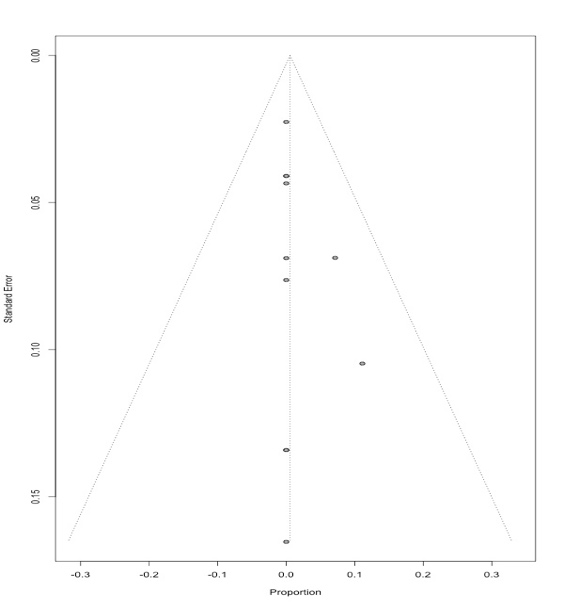 |
| Microscopic transcranial approach | |
| 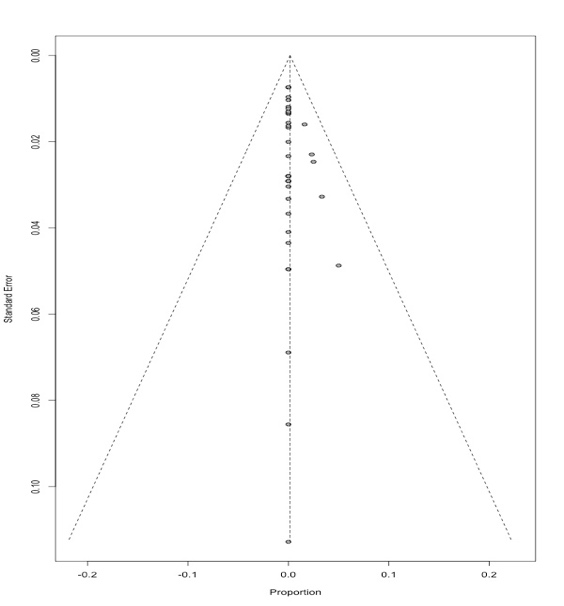 | 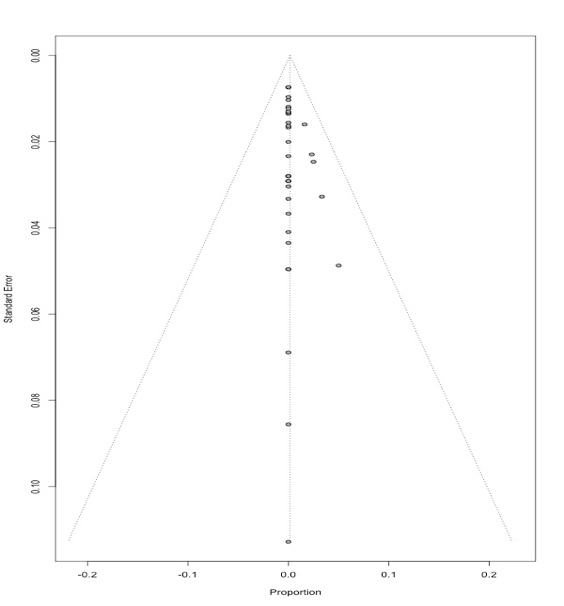 |

| **Olfactory Groove Meningioma** | |
| --- | --- |
| Raw funnel plot | Trim & fill funnel plot |
| Expanded endoscopic endonasal approach | |
| 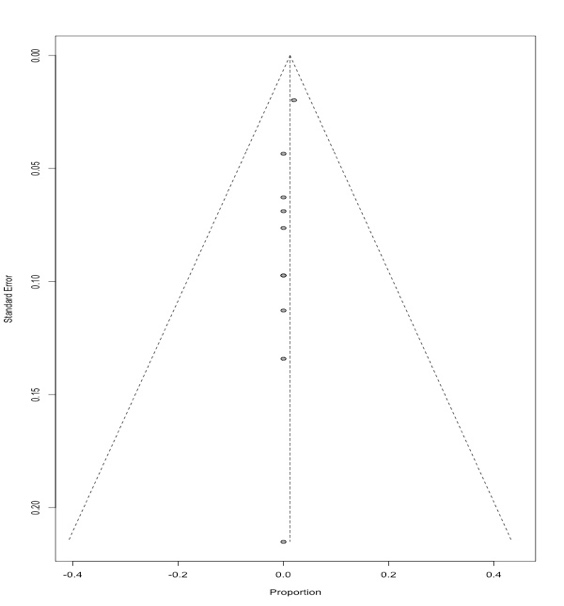 | 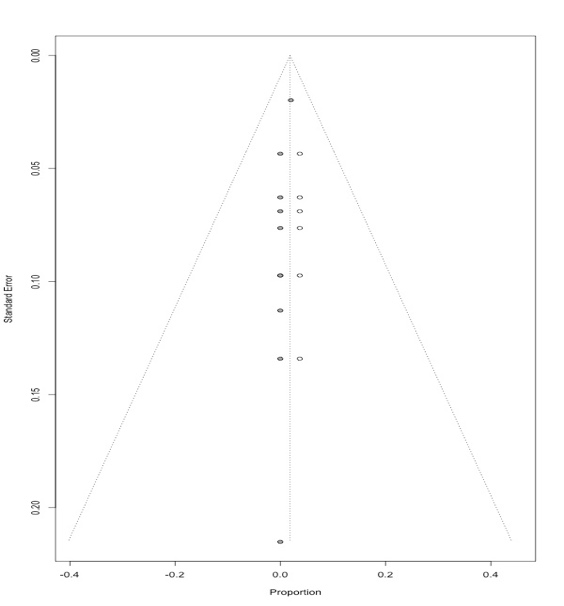 |
| Endoscope-assisted supraorbital keyhole approach | |
| 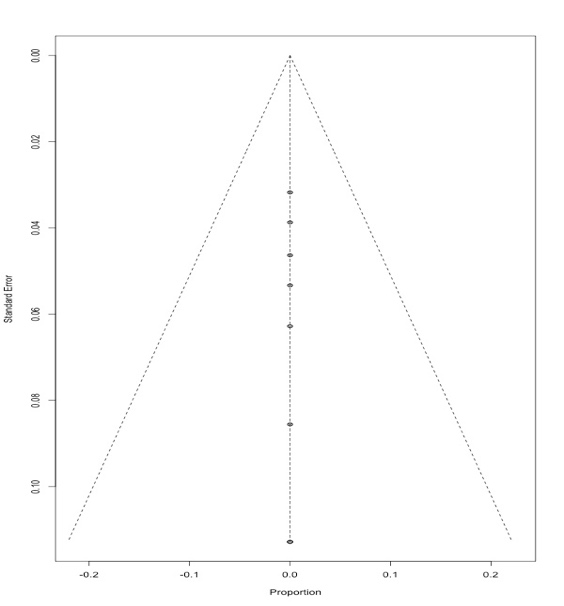 | 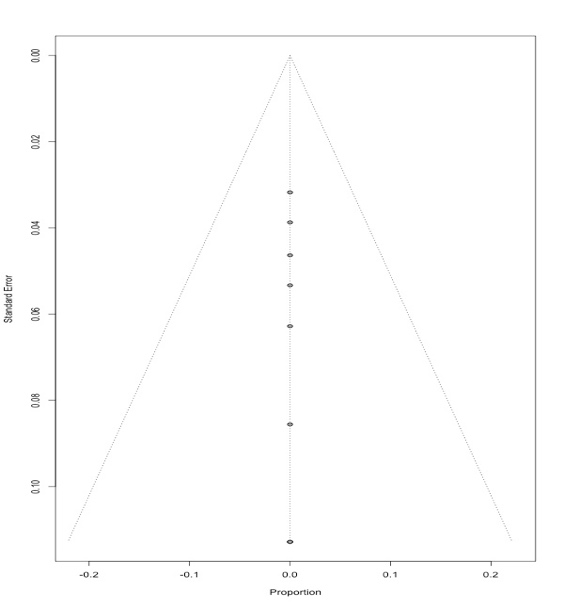 |
| Microscopic transcranial approach | |
| 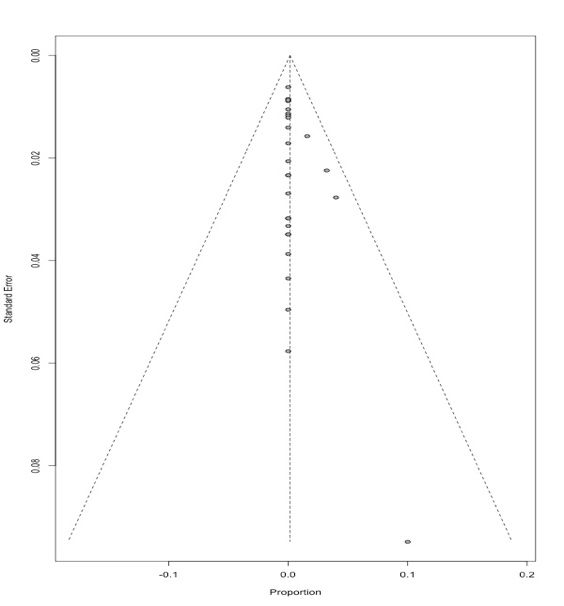 | 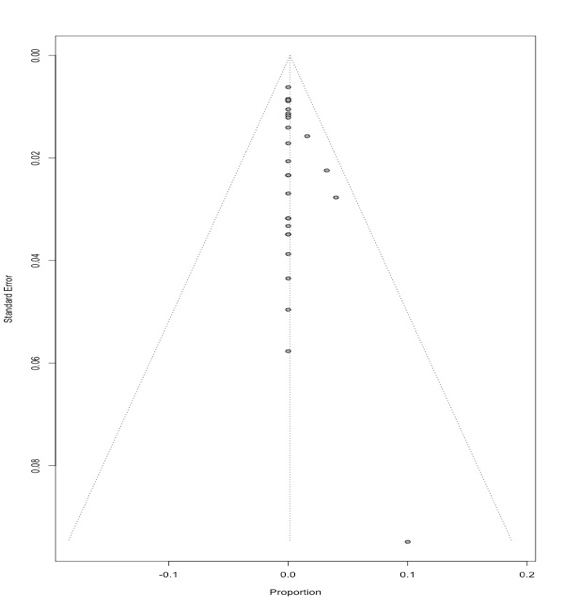 |

**5) 30-day mortality:**

| **Tuberculum Sellae Meningioma** | |
| --- | --- |
| Raw funnel plot | Trim & fill funnel plot |
| Expanded endoscopic endonasal approach | |
| 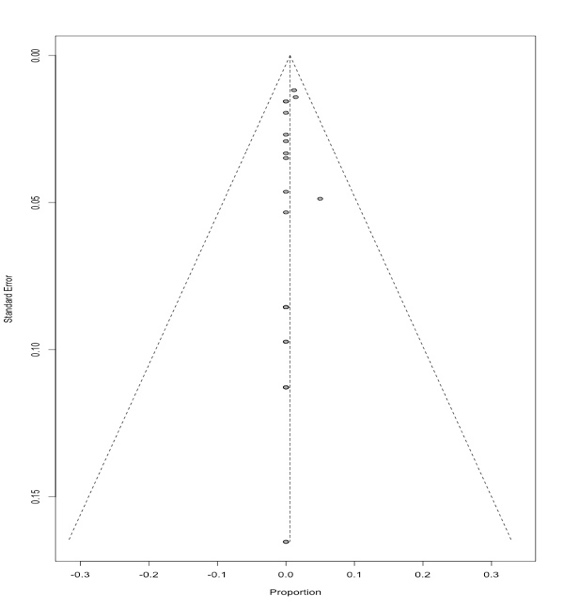 | 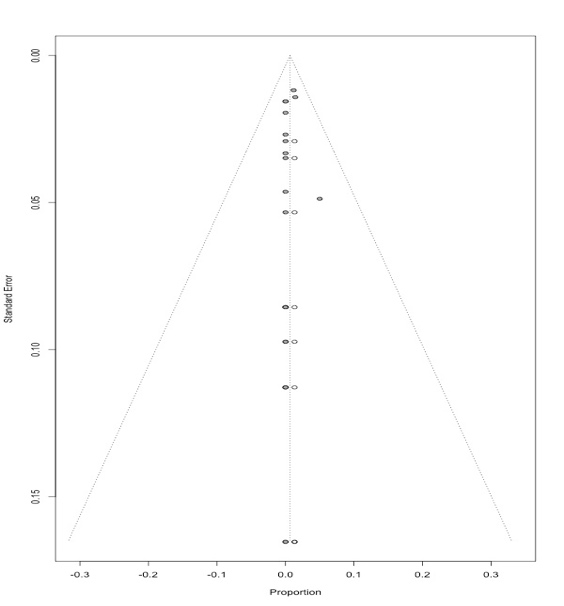 |
| Endoscope-assisted supraorbital keyhole approach | |
| 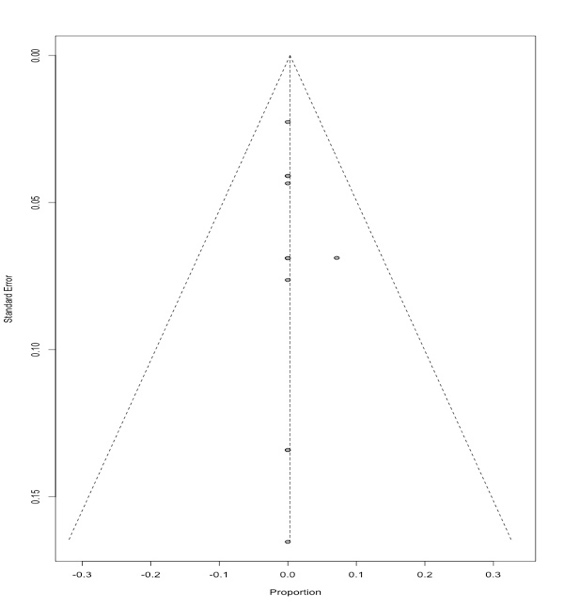 | 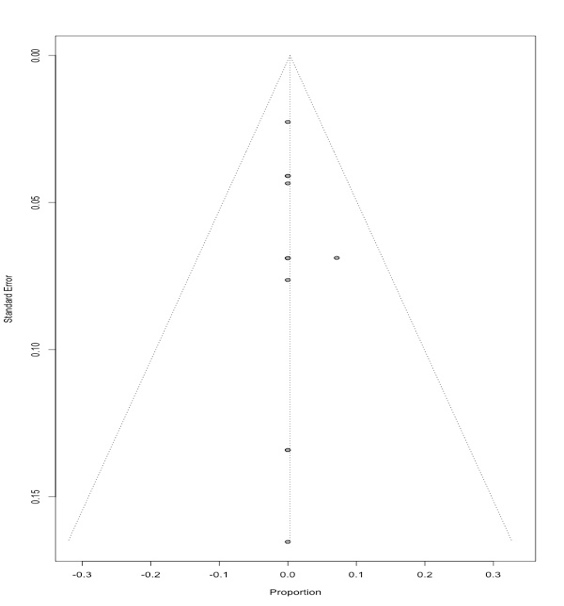 |
| Microscopic transcranial approach | |
| 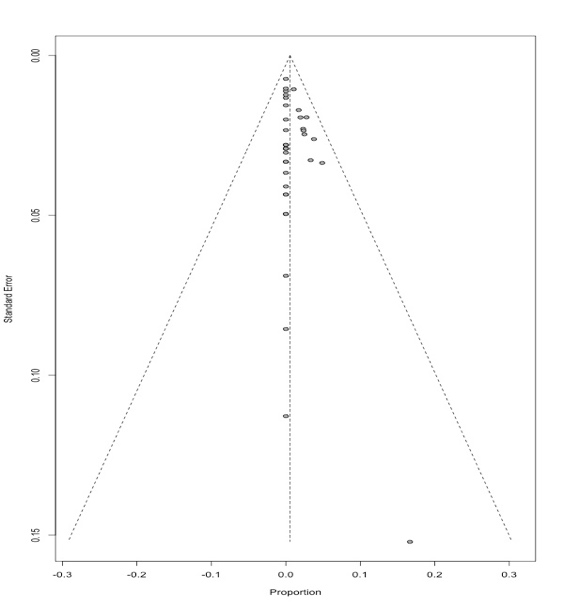 | 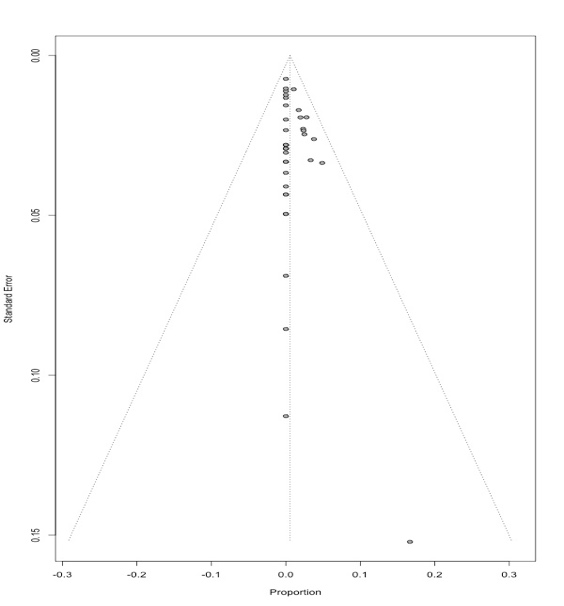 |

| **Olfactory Groove Meningioma** | |
| --- | --- |
| Raw funnel plot | Trim & fill funnel plot |
| Expanded endoscopic endonasal approach | |
| 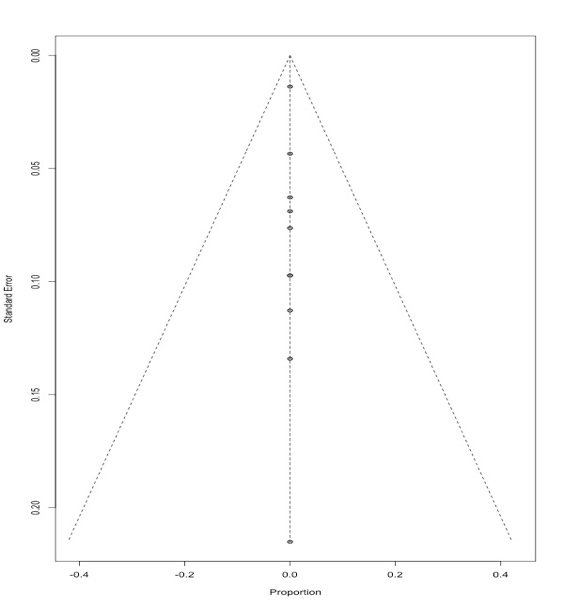 | 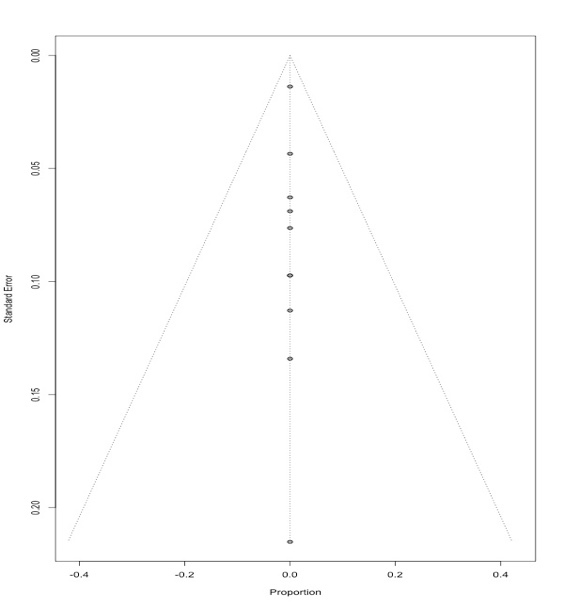 |
| Endoscope-assisted supraorbital keyhole approach | |
| 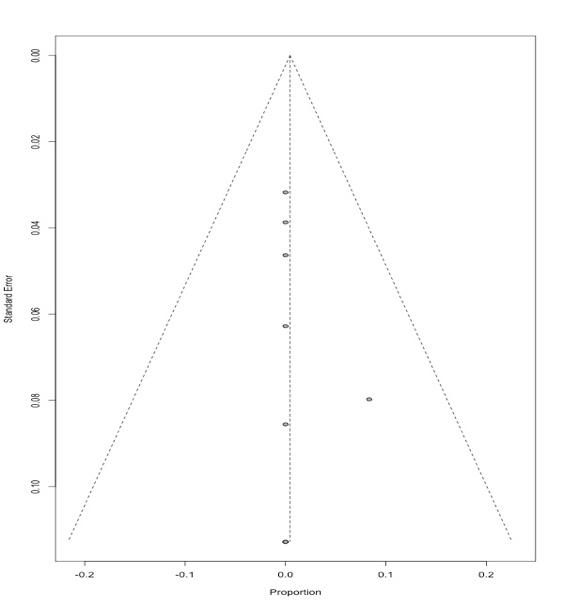 | 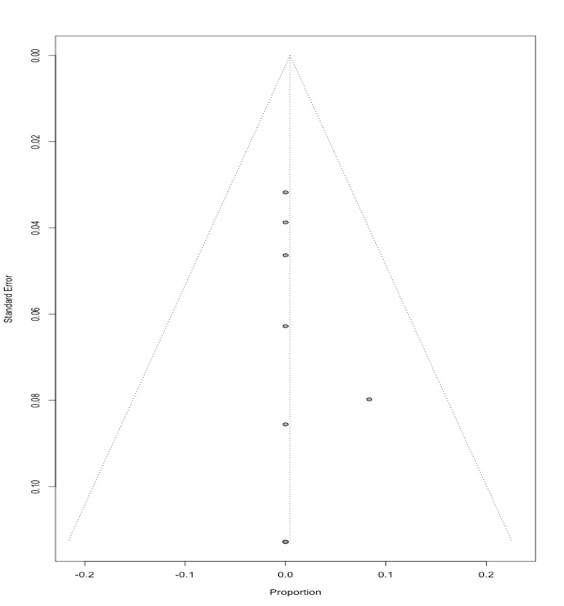 |
| Microscopic transcranial approach | |
| 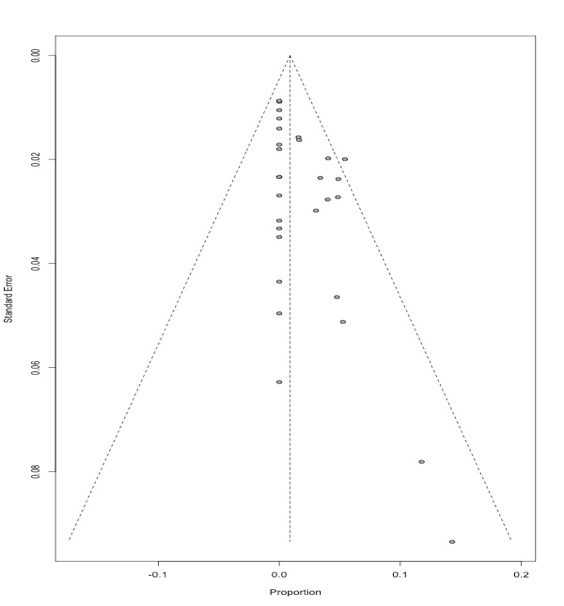 | 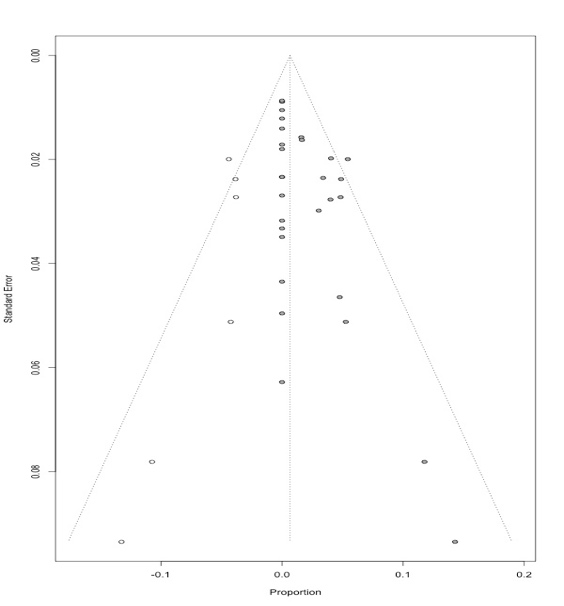 |
