# Supplementary material for: The endoscope-assisted supraorbital “keyhole” approach for anterior skull base meningiomas: an updated meta-analysis
Source: Acta Neurochir (Wien). 2020 Sep 5;163(3):661–76. doi: 10.1007/s00701-020-04544-x (PMC7474310; doi:10.1007/s00701-020-04544-x)
Supplement: Supplementary file 5 — Analysis of low of risk bias studies, compared with overall analysis (DOCX 23 kb) [file 701_2020_4544_MOESM5_ESM.docx]

Table: Analysis of low of risk bias Tuberculum Sellae Meningioma studies, compared to overall analysis. mNOS: modified Newcastle-Ottawa Scale, CI: Confidence intervals. eSKA: Endoscope-assisted supraorbital “keyhole” approach, EEA: Expanded endoscopic endonasal approach, mTCA: Microscopic transcranial approach

| Outcomes in  Tuberculum Sellae Meningiomas | All studies | | Low Bias Risk Studies (mNOS >4/6) | |
| --- | --- | --- | --- | --- |
|  | # of Studies  (# of Cases) | Pooled Incidence % (95% CI) | # of Studies  (# of Cases) | Random effects model  Pooled Incidence % (95% CI) |
| Gross Total Resection (Simpson grade 1 or 2) | | | | |
| EEA | 22 (429) | 83.95 (79.28 – 88.63) | 7 (187) | 80.59 (71.37 – 89.81) |
| eSKA | 10 (112) | 85.21 (73.96 - 96.46) | 7 (89) | 82.08 (67.3 – 96.87) |
| mTCA | 38 (1381) | 89.56 (87.04 – 92.08) | 7 (202) | 87.18 (76.75 – 97.6) |
| Visual improvement | | | | |
| EEA | 19 (366) | 81.98 (71.94 - 92.02) | 6 (162) | 91.19 (83.61 – 98.76) |
| eSKA | 6 (77) | 66.11 (55.7 – 76.53) | 5 (62) | 65.98 (54.4 – 77.56) |
| mTCA | 36 (1280) | 64.9 (57.89 – 71.93) | 8 (301) | 65.5 (51.45 – 79.55) |
| CSF Leak | | | | |
| EEA | 23 (473) | 9.19 (5.33 – 13.05) | 8 (231) | 5.31 (1.82 – 8.81) |
| eSKA | 9 (108) | 2.11 (0 – 5.84) | 7 (89) | 2.67 (0 – 6.87) |
| mTCA | 32 (1182) | 1.58 (0.68 – 2.48) | 9 (312) | 0.33 (0 – 1.47) |
| Intra-operative Arterial Injury | | | | |
| EEA | 22 (426) | 0.26 (0 – 1.34) | 8 (207) | 0.04 (0 – 1.37) |
| eSKA | 11 (128) | 0.56 (0 – 3.48) | 7 (89) | 0.77 (0 – 4.2) |
| mTCA | 32 (1262) | 0.16 (0 - 0.72) | 10 (325) | 0.15 (0 – 1.11) |
| 30-day Mortality | | | | |
| EEA | 23 (471) | 0.6 (0 – 1.74) | 9 (251) | 0.46 (0 – 1.9) |
| eSKA | 11 (128) | 0.33 (0 - 3.21) | 7 (89) | 0.44 (0 – 3.81) |
| mTCA | 37 (1283) | 0.56 (0 – 1.2) | 10 (325) | 0.42 (0 – 1.15) |

Table: Analysis of low of risk bias Olfactory Groove Meningioma studies, compared to overall analysis. mNOS: modified Newcastle-Ottawa Scale, CI: Confidence intervals.

| Outcomes in  Olfactory Groove Meningiomas | All studies | | Low Bias Risk Studies (mNOS >4/6) | |
| --- | --- | --- | --- | --- |
|  | # of Studies  (# of Cases) | Pooled Incidence % (95% CI) | # of Studies  (# of Cases) | Random effects model  Pooled Incidence % (95% CI) |
| Gross Total Resection (Simpson grade 1 or 2) | | | | |
| EEA | 9 (100) | 82.78 (72.3 – 93.26) | 4 (22) | 88.1 (69.9 – 100) |
| eSKA | 8 (75) | 84.9 (50.42 – 100) | 5 (39) | 95.69 (87.42 - 100) |
| mTCA | 28 (1295) | 91.08 (87.91 – 94.24) | 9 (358) | 91.13 (84.97 – 97.3) |
| Visual improvement | | | | |
| EEA | 6 (30) | 54.56 (20.4 – 88.73) | 2 (5) | 50.95 (0 – 100) |
| eSKA | 2 (12) | 52.93 (0 - 100) | 2 (12) | 52.93 (0 - 100) |
| mTCA | 11 (236) | 45.71 (24.54 – 66.88) | 3 (69) | 45.32 (8.92 – 81.71) |
| CSF Leak | | | | |
| EEA | 9 (109) | 14.46 (4.82 – 24.1) | 5 (39) | 2.94 (0 – 100) |
| eSKA | 7 (58) | 1.61 (0 – 7.27) | 4 (22) | 4.39 (0 – 15.58) |
| mTCA | 26 (1132) | 6.45 (3.95 – 8.95) | 8 (324) | 9.37 (2.56 – 16.19) |
| Intra-operative Arterial Injury | | | | |
| EEA | 10 (115) | 1.25 (0 – 4.32) | 5 (28) | 0 (0 – 8.85) |
| eSKA | 9 (96) | 0 (0-3.52) | 5 (39) | 0 (0 – 6.54) |
| mTCA | 27 (1142) | 0.15 (0-0.7) | 9 (337) | 0.42 (0 – 1.6) |
| 30-day Mortality | | | | |
| EEA | 23 (471) | 0 (0 - 2.38) | 5 (28) | 0 (0 – 8.58) |
| eSKA | 9 (96) | 0.45 (0 – 4.09) | 5 (39) | 1.86 (0 – 9.24) |
| mTCA | 10 (115) | 0.89 (0.21 – 1.57) | 10 (375) | 0.45 (0 – 1.58) |
